# The AGC Kinase YpkA Regulates Sphingolipids Biosynthesis and Physically Interacts With SakA MAP Kinase in *Aspergillus fumigatus*

**DOI:** 10.3389/fmicb.2018.03347

**Published:** 2019-01-14

**Authors:** João Henrique Tadini Marilhano Fabri, Naiane Lima Godoy, Marina Campos Rocha, Mansa Munshi, Tiago Alexandre Cocio, Marcia Regina von Zeska Kress, Taicia Pacheco Fill, Anderson Ferreira da Cunha, Maurizio Del Poeta, Iran Malavazi

**Affiliations:** ^1^Departamento de Genética e Evolução, Centro de Ciências Biológicas e da Saúde, Universidade Federal de São Carlos, São Carlos, Brazil; ^2^Department of Molecular Genetics and Microbiology, Stony Brook University, Stony Brook, NY, United States; ^3^Departamento de Análises Clínicas Toxicológicas e Bromatológicas, Faculdade de Ciências Farmacêuticas de Ribeirão Preto, Universidade de São Paulo, Ribeirão Preto, Brazil; ^4^Instituto de Química, Universidade Estadual de Campinas, Campinas, Brazil; ^5^Division of Infectious Diseases, School of Medicine, Stony Brook University, Stony Brook, NY, United States; ^6^Institute of Chemical Biology and Drug Discovery, Stony Brook University, Stony Brook, NY, United States; ^7^Veterans Administration Medical Center, Northport, NY, United States

**Keywords:** *Aspergillus fumigatus*, sphingolipids, MpkA, SakA, YpkA

## Abstract

Sphingolipids (SL) are complex lipids and components of the plasma membrane which are involved in numerous cellular processes, as well as important for virulence of different fungal pathogens. In yeast, SL biosynthesis is regulated by the “AGC kinases” Ypk1 and Ypk2, which also seem to connect the SL biosynthesis with the cell wall integrity (CWI) and the High Osmolarity Glycerol (HOG) pathways. Here, we investigate the role of *ypkA*^Y PK1^ in SL biosynthesis and its relationship with the CWI and the HOG pathways in the opportunistic human pathogen *Aspergillus fumigatus*. We found that *ypkA* is important for fungal viability, since the Δ*ypkA* strain presented a drastically sick phenotype and complete absence of conidiation. We observed that under repressive condition, the conditional mutant *niiA::ypkA* exhibited vegetative growth defects, impaired germination and thermosensitivity. In addition, the *ypkA* loss of function caused a decrease in glycosphingolipid (GSL) levels, especially the metabolic intermediates belonging to the neutral GSL branch including dihydroceramide (DHC), ceramide (Cer), and glucosylceramide (GlcCer), but interestingly a small increase in ergosterol content. Genetic analyzes showed that *ypkA* genetically interacts with the MAP kinases of CWI and HOG pathways, *mpkA* and *sakA*, respectively, while only SakA physically interacts with YpkA. Our results suggest that YpkA is important for fungal survival through the regulation of GSL biosynthesis and cross talks with *A. fumigatus* MAP kinase pathways.

## Introduction

*Aspergillus fumigatus* is a saprophytic filamentous fungus found ubiquitously in soil where it plays a key role in nutrient recycling (Dagenais and Keller, 2009; Perez-Nadales et al., 2014). This organism is also an opportunistic human pathogen that causes invasive pulmonary aspergillosis (IPA), a disease associated with exceptional high mortality rates (50–90%) in the susceptible population (Brown et al., 2012a,b). During the infection, *A. fumigatus* conidia inhaled by the mammalian host can germinate into hyphae within the lung tissue, subsequently colonize the epithelial layer and eventually disseminate to other organs [reviewed in Abad et al. (2010)].

Plasma membrane has long been targeted for antifungal chemotherapy. Amphotericin B (AMB) and azoles such as voriconazole are drugs that disrupt cell membrane integrity (Beauvais and Latge, 2001; Walsh et al., 2008) and stand as part of the first line of IPA therapy (Patterson et al., 2016). However, AMB is notoriously known by its harsh toxicity (Fanos and Cataldi, 2000; Shapiro et al., 2011), while long-term azole therapies can favor the emergence of resistant clinical isolates (Bellete et al., 2010; Camps et al., 2012; Lelievre et al., 2013). In addition, since the last decade, azole-resistant environmental isolates have been documented worldwide (Snelders et al., 2008; Howard et al., 2009; Bader et al., 2015).

Sphingolipids (SL) are a group of complex lipids present in eukaryotic plasma membrane. In yeasts and filamentous fungi, they are involved in pivotal cellular processes such as heat stress tolerance, endocytosis, apoptosis, and polar growth (Dickson et al., 1997; Jenkins et al., 1997; Zanolari et al., 2000; Cheng et al., 2003). SL also associate with sterols in eukaryotic membranes to form microdomains known as lipid rafts, which are critical for signal transduction and membrane protein trafficking [reviewed in Alvarez et al. (2007) and Rella et al. (2016)].

In fungal cells, the endoplasmic reticulum (ER) resident enzyme serine palmitoyltransferase (SPT) catalyzes the first step of SL biosynthesis, condensing serine and palmitoyl coenzyme A to form the 18 carbon intermediate. This molecule (3-keto dihydrosphingosine) is the initial SL precursor that after additional enzymatic steps ends up in the long non-polar sphingoid base chain, also called long chain base (LCB) (Singh and Del Poeta, 2016; Fernandes et al., 2018). SL therefore consist of a LCB attached to a fatty acid via an amide bond with the 2-amino group and to a polar group at the C1 position via an ester bond (Heung et al., 2006). Different carbohydrate groups can modify the LCB C1 position thus producing different types of SL (Heung et al., 2006). The most common fungal SL are the glycosphingolipids (GSL) that comprises two major classes, i.e., the neutral SL such as glucosylceramide (GlcCer) and galactosylceramide (GalCer) and the acidic SL that include the inositolphosphorylceramides (IPCs), mannosyl-inositol phosphoylceramide (MIPC), mannosyl-diinositolphosphorylceramide (MIP_2_C) and others [reviewed in Fernandes et al. (2018)]. These molecules are structurally dissimilar from the mammalian counterparts and therefore attractive antifungal drug targets (Rollin-Pinheiro et al., 2016; Singh and Del Poeta, 2016; Lazzarini et al., 2018). In pathogenic fungi, genetic impairment of GSL biosynthesis is associated with compromised growth, differentiation and virulence (Luberto et al., 2001; Mille et al., 2004; Shea et al., 2006; Singh et al., 2012). For instance, IPC and GlcCer are required for the virulence of *Candida albicans* and *Cryptococcus neoformans* (Zhong et al., 2000; Rittershaus et al., 2006; Singh et al., 2012; Munshi et al., 2018). In addition, SL are also important for sustaining growth and virulence of plant pathogens (Ramamoorthy et al., 2007, 2009; Zhu et al., 2014).

In *Saccharomyces cerevisiae*, the *de novo* SL biosynthesis is regulated by the paralogs Ypk1 and Ypk2 protein kinases, two members of the AGC kinase subfamily (Roelants et al., 2002; Niles et al., 2012). These kinases are homologs of the mammalian serum and glucocorticoid-regulated kinase (SGRK) and comprise the less studied enzymes of this family (Pearce et al., 2010). Ypk1/2 subcellular localization and phosphorylation are regulated by the intracellular SL level (Sun et al., 2000). Ypk1/2 are phosphorylated by the upstream kinases Pkh1 and Pkh2 (homologs of mammalian PDK), localized at or adjacent to the plasma membrane, and also by the Torc2 kinase (Roelants et al., 2002; Kamada et al., 2005; Niles et al., 2012). Following activation, Ypk1/2 phosphorylate the inhibitory regulators Orm1 and Orm2 located at the ER membrane, which in turn activate SPT, thus causing an increase in SL levels (Roelants et al., 2011).

In *A. fumigatus*, the highly conserved mitogen-activated protein kinase (MAPK) signaling pathways, coordinated by MpkA^MPK1^ and the paralogs SakA^HOG1^ and MpkC, are essential for the adaptation to different stresses which therefore govern the expression of several virulence determinants such as cell wall composition, secondary metabolites production, tolerance to antifungals and osmotic stress (Jain et al., 2011; Altwasser et al., 2015; Bruder Nascimento et al., 2016; Macheleidt et al., 2016). In yeast, Ypk1/2 connect the SL biosynthesis and the cell wall integrity (CWI) pathway (Roelants et al., 2002; Schmelzle et al., 2002). Besides phosphorylating Ypk1/2, yeast Pkh1/2 can also phosphorylate and activate the Protein Kinase C (Pkc1) (Inagaki et al., 1999), which activates the CWI pathway enabling the activity of Mpk1 MAP kinase [reviewed in Levin (2011)]. Moreover, Ypk1/2 maintain the Rho1 GTPase positioned at the plasma membrane, allowing Pkc1 activation in response to membrane stress and assisting the actin cytoskeleton reorganization (Hatakeyama et al., 2017). Likewise, the SL pathway regulates Pkc1 in *C. neoformans* (Heung et al., 2004). Thus, SL biosynthesis and the CWI pathway in fungi may intersect through the complete activation of Pkc1 by Pkh1 and/or Pkh2, and through Ypk1 and/or Ypk2 (Schmelzle et al., 2002). In addition, reports have pointed out to a relationship between the yeast High Osmolarity Glycerol (HOG) pathway, regulated by Hog1 MAP kinase, and the SL metabolism (Barbosa et al., 2012; Yamaguchi et al., 2017). For instance, the yeast HOG pathway is activated during inhibition of SL and ergosterol biosynthesis (Tanigawa et al., 2012) playing a protective role against the growth defect caused by impaired SL biosynthesis (Yamaguchi et al., 2017).

Although genetic evidence indicates that Ypk1 homolog is implicated in SL production in *A. nidulans* (Colabardini et al., 2013), the role of *ypkA*^Y PK1^ homolog in *A. fumigatus* has not been studied and no information is available if the YpkA signaling cascade cross talks with *A. fumigatus* MAPK pathways. Here, we investigate the function of *A. fumigatus ypkA* verifying its importance for fungal growth, SL biosynthesis and its interaction with the CWI and HOG pathways.

## Materials and Methods

### Strains and Culture Conditions

The *A. fumigatus* strains used in this study are described in Supplementary Table S1. Strains were maintained in complete medium [YG; glucose 2% (w/w), 0.5% yeast extract (w/w), 1× trace elements)] or minimal medium [MM; glucose 1% (w/w), 1× high-nitrate salts, 1× trace elements, pH 6.5)]. Trace elements and high nitrate salt compositions were as described previously (Kafer, 1977; Malavazi and Goldman, 2012). To grow the *niiA::ypkA* strain, modified minimal medium was used [AMM; glucose 1% (w/w), 2× salt solution, 1× trace elements, pH 6.5]. The composition of the salt solution (without nitrate) and the trace elements solution for AMM was described previously (Cove, 1966). The induction and repression of *niiA* conditional promoter was achieved by using AMM supplemented with 10 mM of magnesium nitrate (MN) or 50 mM of ammonium tartrate (AT), respectively, as described elsewhere (Hu et al., 2007). For solid media, 2% agar (w/w) was added. To grow the ΔKU80 pyrG1 strain, the media was supplemented with 1.2 g/l of uridine and uracil. When required, pyrithiamine (Sigma) was added to a final concentration of 0.2 μg/ml.

To assess the germination kinetics of the *niiA::ypkA* strain, 1 × 10^6^ conidia of each strain were inoculated onto glass coverslips that were placed in 35 mm Petri dishes containing 2 ml of AMM supplemented with MN or AT and were incubated at 37°C for 2, 4, 6, and 8 h. After incubation, the coverslips with adherent germlings were transferred to fixative solution [PBS 1×, DMSO 5% (v/v) and formaldehyde 3.7% (v/v)] for 10 min. The coverslips were rinsed with PBS 1×, mounted and visualized under a bright field microscope. A conidia was counted as germinated if a germ tube was evident.

To evaluate the *ypkA* expression in the conditional mutant by RT-qPCR, 2 × 10^7^ conidia of *niiA::ypkA* mutant strain were incubated in 50 ml of liquid AMM + AT (37°C) for 24 h. The mycelia were then transferred to fresh pre-heated media AMM + AT or AMM + MN for additional 6 h at 37°C. Heat shock stress induction for immunoblot analysis of SakA and MpkA phosphorylation were achieved by incubating 1 × 10^8^ conidia from wild-type and *niiA::ypkA* mutant strain in 50 ml of liquid AMM + AT for 24 h at 30°C. Subsequently, heat shock was induced by transferring the mycelia to fresh pre – heated AMM + AT (48°C) for additional 15, 30, and 60 min of incubation at 48°C. The control was left at 30°C. The same procedures were employed to induce heat shock on the wild-type, Δ*mpkA*, Δ*sakA*, and *sakA*::GFP *ypkA*::3xHA strains, however, using MM for 24 h prior heat shock for 5, 15, 30, and 60 min at 48°C. Mycelia from each time point, both pre- and post-heat shock, were collected via vacuum filtration, frozen in liquid nitrogen and stored at -80°C until used for RNA or protein extractions.

### Construction of the *A. fumigatus* Mutants

All the gene replacement cassettes were constructed by *in vivo* recombination in *S. cerevisiae* as reported previously (Malavazi and Goldman, 2012). The Δ*ypkA*, *niiA::ypkA*, *ypkA*::GFP, and *ypkA*::3xHA were generated using the primers and the strategies described in Supplementary Table S2 and Supplementary Figure S1.

To generate the double mutant Δ*mpkA niiA::ypkA*, the *mpkA* deletion cassette was amplified from the genomic DNA of the Δ*mpkA* strain (Valiante et al., 2009) using primers MpkA 5 FW and MpkA 3 REV and transformed into the *niiA::ypkA* strain. The *mpkA* replacement was checked by using the primers mpkA 600 ups and MpkA 3′ REV, as well as mpkA FW and mpkA REV (Supplementary Table S2 and Supplementary Figures S1E,F, respectively). Likewise, to construct the double mutant Δ*sakA niiA::ypkA*, the *sakA* deletion cassette was amplified from the genomic DNA of the Δ*sakA* strain (Altwasser et al., 2015) using primers SakA yes FOR and SakA yes REV and transformed into the *niiA::ypkA* strain. The *sakA* replacement was checked by using the primers SakA 500 ups and SakA yes REV and sakA pET15b part 2 FW and sakA pET15b part 3 REV (Supplementary Figures S1G,H, respectively).

To generate the double tagged *sakA::*GFP *ypkA::*3xHA strain, the *ypkA*::3xHA cassette (Supplementary Figure S1L,M) was amplified from the pRS426 plasmid harboring the recombined cassette using primers ypkA 1400 FW and Afu2g10620 3R and transformed into the *sakA::*GFP strain (Bruder Nascimento et al., 2016). The strain was validated by PCR using the primers ypkA 500 ups and prtA REV (Supplementary Figure S1O), and by Western blot analysis using α-HA and α-GFP antibodies (Supplementary Figures S1P,Q).

### DNA Manipulation and Southern Blot Analysis

Southern blot analysis was used to confirm that the *niiA::ypkA* cassette integrated homologously at the targeted locus. Genomic DNA from *A. fumigatus* was extracted as previously described (Malavazi and Goldman, 2012). For Southern blot analysis, *Apa*I-restricted chromosomal DNA fragments were separated on a 1% agarose gel and blotted onto Hybond N^+^ nylon membranes (GE Healthcare), following standard techniques (Sambrook and Russell, 2001). Probe labeling for detection was performed using AlkPhos Direct Labeling and Detection System (GE Healthcare) according to the manufacturer’s description. Labeled membranes were exposed to Hyperfilm ECL (GE Healthcare) and were scanned to image processing.

### Staining and Microscopy

To analyze hyphal growth and conidiophore formation, wild-type and Δ*ypkA* strains were grown and analyzed as described in Rocha et al. (2016). Filipin, a fluorescent polyene antibiotic that binds sterols (Alvarez et al., 2007), was used to evaluate sterol distribution in the germlings. Accordingly, 1 × 10^5^ conidia of wild-type and *niiA::ypkA* strains were inoculated in 2 ml of AMM supplemented with MN or AT in glass bottom dishes (MatTek Corporation) at 37°C for 12 h. Subsequently, hyphae were stained with 25 μg/ml of filipin for 5 min in preheated AMM (37°C). Coverslips were washed with AMM and analyzed. Conidia of *ypkA*::GFP strain were cultivated and processed as described above, however, using MM at 30°C. Glass bottom dishes containing adherent germilings were exposed to 45°C for 20 or 30 min do induce heat shock and immediately analyzed.

For all conditions, germlings were analyzed on Observer Z1 fluorescence microscope (Carl Zeiss). Filipin and GFP were visualized using 49 and 38 HE filter sets (Carl Zeiss), respectively, and 100× magnification oil immersion objective (Plan-Apochromat; NA 1.4). DIC (Differential Interference Contrast) and fluorescent images were captured with an AxioCam camera (Carl Zeiss) and processed using AxioVision software.

### Phenotypic Assays

The growth rate was determined at different temperatures by spotting 1 × 10^5^ conidia into the center of a 90 mm Petri dish containing 20 ml of solid AMM supplemented with MN or AT. The diameter was scored at 24-h intervals. The susceptibility of mutant strains to agents that impair maintenance of cell wall or cell membrane [caffeine (CAF), Calcofluor White (CFW), Congo Red (CR), and Sodium Dodecyl Sulfate (SDS), respectively], or inhibit SL or ergosterol biosynthesis [myriocin (MYR) and lovastatin (LOV), respectively] was evaluated by assessing the initial growth of conidia from a serial dilution. Drop out experiments were performed by spotting 10-fold dilution series on different growth media, supplemented with the drugs and AT or MN, when necessary. Alternatively, susceptibility to aureobasidin A (ABA) and cerulenin (CRN), inhibitors of GSL and fatty acid synthesis, respectively, was determined by using an inoculum of 1 × 10^4^ conidia grown on 200 μl of solid AMM supplemented with MN or AT. The plates were incubated for 48 h at 37°C and analyzed.

### RNA Extraction and Gene Expression Procedures

Mycelia were disrupted by grinding in liquid nitrogen. The total RNA was extracted with Trizol reagent (Thermo Fisher Scientific) according to the manufacturer’s protocol. RNA was processed as described previously (Rocha et al., 2015). Briefly, the samples were treated with Turbo DNase I (Thermo Fisher Scientific) and the RNA integrity was assessed with a 2100 Bioanalyzer (Agilent Technologies). DNAse-treated total RNA from each strain (2 μg) was reverse-transcribed with High Capacity cDNA Reverse Transcription kit (Thermo Fisher Scientific) using oligo dTV and random primers blend. RT-qPCR was conducted with a Power Sybr Green PCR Master Mix (Thermo Fisher Scientific). The primers for the individual genes were designed using Primer Express 3.0 software (Life Technologies) and are listed in Supplementary Table S3. RT-qPCR was performed in duplicate from three independent biological samples in a StepOne Plus Real Time PCR System (Thermo Fisher Scientific). The fold change in mRNA abundance was calculated using 2^-ΔΔCt^ (Livak and Schmittgen, 2001) and all the values were normalized to the expression of the *A. fumigatus* β-tubulin (*tubA*). Statistical analysis was performed using one-way ANOVA with Tukey’s *post hoc* test (*p* ≤ 0.05).

### Ergosterol Extraction and Quantification by HPLC-UV

**Figure 1:**
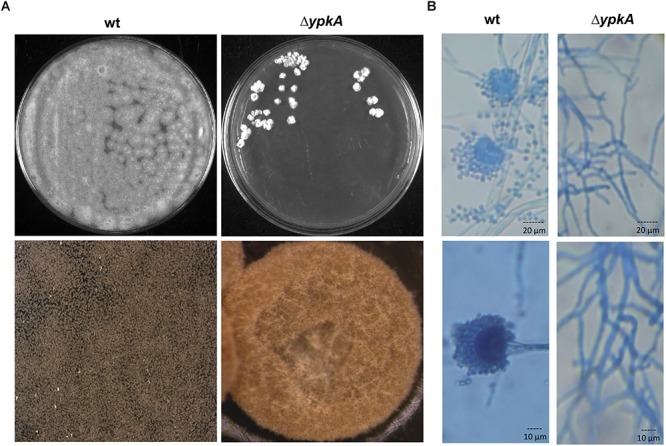
*ypkA* deletion impairs the vegetative growth and conidiation. **(A)** Growth phenotypes of wild-type strain and Δ*ypkA* mutant after 120 h of growth in YG medium at 37°C (upper panel). Growth rate is compromised and asexual conidiation is abolished in the Δ*ypkA* strain. Lower panel: 20× magnification of colonies. **(B)** Hyphae and conidiophores morphology of the wild-type and Δ*ypkA* mutant strain after 120 h of cultivation on solid YG. Slide cultures and lactophenol cotton blue staining reveal abnormal hyphae and absence of asexual reproductive structures and conidia (40 and 100× magnification, respectively).

The biomass of wild-type and the conditional *niiA::ypkA* strain were obtained by growing 1 × 10^7^ conidia in AMM supplemented with MN or AT for 24 h at 37°C. Due to the absence of conidiation and the drastically sick phenotype of the Δ*ypkA* deletion mutant (see Figure 1), the biomass was obtained as following: 1 × 10^3^ conidia of wild-type strain and five microcolonies (approximately 3–5 mm) from a 5-days culture on solid YG + 0.1% casamino acids were directly inoculated in YG at 37°C. The glucose utilization was used as a parameter to normalize growth between the two cultures. Glucose consumption was determined by Glucose Oxidase Assay Kit K-GLOX (Megazyme), following the manufacturer’s instructions. Equal glucose consumption between the strains was achieved after 120 h of incubation, when growth was interrupted.

For all conditions, the biomass was collected by vacuum filtration and rinsed with distilled water. Samples were frozen in liquid nitrogen, lyophilized and stored at -80°C until use. Ergosterol extraction was done as described elsewhere with few modifications (Anderson et al., 1994). Briefly, 20 mg of each sample were mixed with 500 μl of methanol in an ultrasonic bath for 30 min at a constant temperature of 50°C. The mixture was subsequently centrifuged at maximum speed for 5 min and filtered through a 0.22 μm PTFE filter and 10 μl samples of each fraction were injected for HPLC-UV analysis. The extractions and analysis were performed in quadruplicate.

The HPLC system consisted of a separation module (Waters Alliance 2695), equipped with the software MassLynx 4.1 v (Waters) and a quaternary pump, an in-line vacuum degasser and a photodiode array detector. Chromatographic separation was performed on a phenyl-hexyl column (250 mm × 4 mm, 5 mm, Phenomenex) using a linear gradient elution of acetonitrile and water, which were both supplemented with 0.1% formic acid, from 80 to 100%, over 20 min. The ergosterol peaks were identified based on the retention time (λ_282 nm_).

### Extraction of *A. fumigatus* Lipids

The lipids extraction was performed from the same mycelia obtained for ergosterol quantification. The extraction was followed as described in Munshi et al. (2018) with minor modifications. Briefly, 50 mg of wild-type and *niiA::ypkA* mycelia were transferred to glass tubes and 2 ml of Mandala buffer was added along with approximately 1 ml of glass beads. These tubes were then vortexed vigorously for 1 min and extraction was followed as mentioned in Mandala et al. (1995). This was followed by Bligh and Dyer extraction (Bligh and Dyer, 1959) and base hydrolysis (Clarke and Dawson, 1981). After Bligh and Dyer extraction, 1/4th of the total volume of sample was removed for phosphate estimation. At the end of the base hydrolysis, samples were dried in a nitrogen evaporator (Organomation N-EVAP^TM^ 112). Internal standards were added externally during the lipidomic analysis. Experiments were run using three independent biological repetitions.

### Sphingolipids Mass Spectrometry Analysis

Mass spectrometry analysis was carried out as mentioned in Munshi et al. (2018). Briefly, a Thermo Accela HPLC system (San Jose, CA, United States) was used to separate the dried extracts dissolved in 150 μl of ammonium formate (1 mM) with 0.2% of formic acid in methanol. Peeke Scientific Spectra C8 (Redwood City, CA, United States) HPLC column (150 mm × 3 mm) was used into which 10 μl of samples were injected. The buffers used for the runs were as follows: buffer A [2 mM ammonium formate and 0.2% formic acid (FA)] and buffer B, [1 mM ammonium formate and 0.2% FA in methanol]. A gradient using buffer A and B was used, starting with 70% B with an increase to 90% over 5 min, followed by a ramp to 99% B over 9 min. The column was equilibrated with initial conditions for 8 min at a flow rate of 500 μl/min. The HPLC was coupled to the HESI source of a Thermo TSQ Quantum Ultra triple quadrupole mass spectrometer (San Jose, CA, United States). The SL profile was performed using positive ion mode. With the high voltage set to 3.5 kV, vaporizer temperature at 400°C, sheath gas pressure at 60, auxiliary gas pressure at 15 and a capillary temperature of 300°C. The collision cell was operated at 1.5 mTorr of argon. For the duration of the run, transitions for each lipid species were monitored at 100 or 50 ms dwell time. Twenty lipid standards for our profile from Avanti (Alabaster, AL, United States) were used to develop calibration curves and these curves were then used for lipids species to be monitored. Processing of the samples was done using Thermo Xcalibur 2.2 Quan Browser software and exported to excel for reporting results.

To evaluate the differences in SL accumulation in the wild-type and *niiA::ypkA* strains, graphs were plotted with the ratio obtained by dividing SL levels in AMM + AT and AMM + MN (represented as pmol/Pi) for both strains to normalize the inherent growth differences under these two nitrogen sources. All lipid measurements are quantitative analyses using appropriate lipid standards for sphingosine, ceramide, GlcCer and IPC species, which are either commercially available and custom synthesized (e.g., IPC).

### Protein Extraction and Immunoblotting Analysis

To assess the MpkA and SakA phosphorylation, mycelia were obtained upon heat shock stress condition according to the description above. Protein extraction, processing and immunoblotting procedures were performed as described previously (Rocha et al., 2015; Bruder Nascimento et al., 2016).

### Co-immunoprecipitation (Co-IP) Assays With GFP-Trap and α-HA Magnetic Beads

To perform Co-IP assays, the double tagged *sakA*::GFP *ypkA*::3xHA strain was used. For protein extraction, mycelium was grinded in liquid nitrogen. For GFP-Trap Co-IP, about 2 ml of extraction buffer containing 50 mM Tris HCl (pH 7.6), 225 mM KCl, 1 mM sodium orthovanadate, 1% Igepal CA 630 (Sigma), 1 mM PMSF and 1× Complete Mini protease inhibitor (Roche Applied Science) were added to the samples, as described previously (Ries et al., 2016). For Co-IP using Dynabeads protein A, about 2 ml of extraction buffer (B250) containing 100 mM Tris HCl (pH 7.5), 250 mM NaCl, 1 mM EDTA, 1 mM sodium orthovanadate, 0.6 mM benzamidine, 1% glycerol, 0.1% Igepal CA 630 (Sigma), 1 mM PMSF and 1× Complete Mini protease inhibitor were added to the samples, as described previously in Bayram et al. (2008). The extracts were centrifuged at 20,000 *g* for 40 min at 4°C. The supernatants were collected and the protein concentrations were determined according to the Lowry method modified by Hartree (1972).

GFP-Trap Co-IP experiments were performed as previously described (Ries et al., 2016). Briefly, 5 mg of protein were added to 20 μl of GFP-Trap agarose resin (ChromoTek; GTA-20). The resin was centrifuged (2,500 *g* for 2 min at 4°C) and washed three times with resuspension buffer [10 mM Tris HCl (pH 7.5), 150 mM NaCl, 0.5 mM EDTA]. Crude extracts and resin were then incubated with shaking at 4°C (4 h). Subsequently, the resin was spun down for 30 s at 5,000 *g* and washed three times with resuspension buffer. To perform reciprocal Co-IP assays, samples were processed as above following the procedures previously described (Manfiolli et al., 2017). Briefly, 5 mg of protein were added to 20 μl of Dynabeads Protein A (Thermo Fisher Scientific) previously incubated with 4 μl of a mouse monoclonal α-HA antibody (H3663, Sigma) for 30 min with shaking in resuspension buffer (PBS 1×, Tween 20 0.01%), according to the manufacturer’s instructions. Cell extracts and resin were then incubated with shaking at 4°C (2 h). After incubation, the resin was washed three times in B250 buffer by placing the tubes in a DynaMag^TM^ magnet.

For both conditions, to release the proteins from the resin, samples were incubated with 30 μl of 2× Laemmli buffer (Laemmli, 1970) and boiled for 5 min. 20 μl of the immunoprecipitated were run in a 10% SDS-PAGE. Proteins were electroblotted onto a PVDF membrane for Western blot assay. GFP-tagged SakA was detected using a rabbit α-GFP antibody (G1544; Sigma) at 1:1,000 dilution in TBST skimmed milk 3%. For the 3xHA-tagged YpkA detection, a rabbit monoclonal α-HA antibody (3724; Cell Signaling) was used at 1:1,000 dilution in PBST BSA 5%. Secondary α-rabbit IgG horseradish peroxidase (HRP) antibody (A0545; Sigma) in TBST (1:3,000 dilution) was used for a 2-h incubation period at room temperature. Chemoluminescent detection was performed by using an ECL Prime Western Blot detection kit (GE HealthCare). Images were generated by exposing the PVDF membranes to the ChemiDoc XRS gel imaging system (Bio-Rad).

To investigate the interaction between YpkA and MpkA, the YpkA 3xHA immunoprecipitation was processed as described above and analyzed with a rabbit α-phospho p44/42 MAPK antibody (4370; Cell Signaling Technologies). This antibody specifically recognizes *A. fumigatus* MpkA as demonstrated previously (Bruder Nascimento et al., 2016).

## Results

### Identification of the YpkA Homolog in *A. fumigatus*

In *A. nidulans*, two genes named *pkcA* and *pkcB*, which encode two proteins presenting similarity with protein kinase C (PKC), were previously identified (Herrmann et al., 2006). Analysis of *A. fumigatus* genome^1^ using sequences of *A. nidulans* PkcB, yeast Ypk1/2 and serum/glucocorticoid-regulated kinase 2 (SGRK2) from *Homo sapiens* as query sequences revealed that the sequence encoded by the Afu2g10620 gene was the closest homolog. We named this gene as *ypkA* to be consistent with previous *Aspergillus* nomenclature (Colabardini et al., 2013). *A. fumigatus* YpkA shows significant homology with the PkcB (YpkA) of *A. nidulans* (89% identity and 93% similarity, *e*-value 0.0), the yeast Ypk1/Ypk2 (47% identity and 62% similarity, *e*-value 0.0; and 47% identity and 61% similarity, *e*-value 1e-180, respectively) and the human kinase SGRK2 (51% identity and 64% similarity, *e*-value 5e-116). In contrast to yeast, this analysis revealed that *A. fumigatus* possesses a single putative *ypkA*-encoding gene. Additionally, comparative analysis of *A. fumigatus* YpkA and PkcA (Rocha et al., 2015) indicated that similarity is confined to the amino terminal region of the protein (44% identity and 63% similarity, *e*-value 4e-86). This region encompasses residues 296–551 (Protein kinase domain; PFAM PF00069.24) and contains the conserved catalytic site common to all PKCs proteins. Nevertheless, cysteine rich regions, usually involved in binding to diacylglycerol or phorbol esters in classical PKCs, as well as the pseudo-substrate sequence involved in regulating the activity of these PKCs (reviewed in Steinberg, 2008), are absent in this isoform (Supplementary Figure S2). On the other hand, the serine/threonine kinase domain and the ATP binding region are well conserved. Inside the catalytic domain, there are two conserved phosphorylation sites predicted to be phosphorylated by PDK1 and PDK2 enzymes, termed Pkh1 and Pkh2 in yeast. PDK1 site (T451) is located in the activation loop of the catalytic domain while the PDK2 phosphorylation site is distally located (S612) close to the C-terminus of the protein (Supplementary Figure S2) (Casamayor et al., 1999). Importantly, *A. fumigatus* possesses a single PDK homolog gene named *pkhA* in *A. nidulans* (Colabardini et al., 2013). This suggests that if both T451 and S612 phosphorylation sites are functional *in vivo*, they can be presumptive PkhA targets. However, this assumption has not been experimentally addressed in any *Aspergillus* species so far. Taken together, these results indicate that YpkA is a member of the subfamily of AGC protein kinases, so named because it comprises the mammalian protein kinase groups represented by the protein kinase A (PKA), protein kinase G (PKG), protein kinase C (PKC) and also by PKB and the ribosomal kinases S6 (Sobko, 2006).

In addition, the *ypkA* gene architecture described in the AspGD was validated here by RT-PCR and full sequencing indicated that the genomic sequence has 2.294 nucleotides and comprises five introns that interrupt the coding region of 1.914 bp (data not shown). YpkA polypeptide therefore contains 637 residues and calculated molecular weight of 70.9 kDa.

### The *ypkA* Gene Is Essential for Vegetative Growth and Conidiation

In yeast, the double deletion of the *YPK1* and *YPK2* genes is unviable and can be complemented by the human SGRK2 (Casamayor et al., 1999). Since only one *ypkA* copy was found in *A. fumigatus*, we generated a deletion mutant to investigate the role of *ypkA* in the cell membrane homeostasis in *A. fumigatus* (Supplementary Figures S1A,B). Although we were able to obtain viable primary transformants, the Δ*ypkA* strain presented a drastically sick phenotype both in complete or minimal medium, with tiny and irregular shaped colonies without any conidiation (Figure 1A). This phenotype resulted in very slow growth rates both in liquid or solid medium thus affecting the propagation of this strain. These phenotypes were also similar to that previously reported in *A. nidulans* (Colabardini et al., 2013). To further investigate the absence of conidiation in the null mutant, conidiophores stained with lactophenol cotton blue were analyzed by bright field microscopy. Only abnormal hyphae growth was observed in the Δ*ypkA* strain, which did not produce conidiophores and asexual reproductive associated structures (Figure 1B).

We conclude that although growth is severely impaired in this mutant, the hyphae obtained in liquid submerged cultures or solid medium are viable. In fact, aconidial mycelium stocks could be preserved in glycerol 23% at -80°C and revived on complete culture medium.

**Figure 2:**
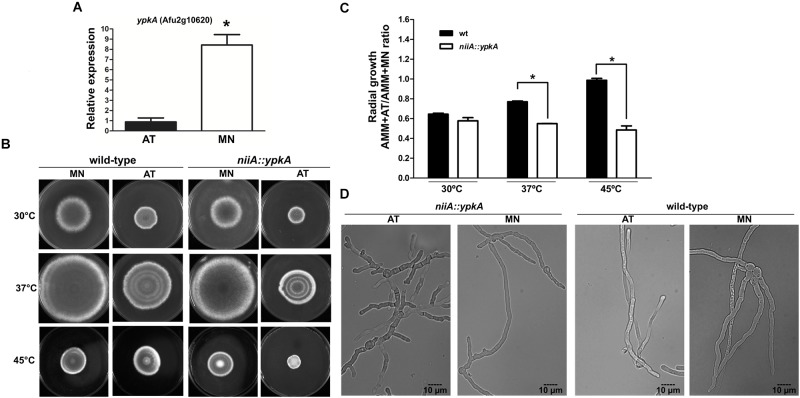
YpkA contributes to vegetative growth and thermo sensitivity. **(A)** Expression of *ypkA* in the *niiA::ypkA* conditional strain, both in repression (AMM + AT) and induction (AMM + MN) conditions. *niiA::ypkA* strain was grown for 24 h in AMM supplemented with 50 mM of AT and transferred to fresh AMM supplemented with 10 mM of MN or 50 mM of AT for 6 h. Fold increase represents the normalized mRNA relative abundance to *niiA::ypkA* strain gown in AMM + AT. **(B,C)** 1 × 10^5^ conidia of each strain were inoculated on solid AMM + MN or AMM + AT and radial growth was measured after 5 days at the indicated temperatures. The graph shows the ratio obtained by dividing growth values in AMM + AT and AMM + MN to normalize the inherent growth differences under these two nitrogen sources observed for the wild-type strain. **(D)** 1 × 10^5^ conidia of each strain were inoculated in 2 ml of liquid AMM + AT or AMM + MN and incubated at 37°C during 12 h before analysis in bright field microscope equipped with DIC. Average ± SD (*n* = 3) are shown (^∗^*p* ≤ 0.05, Student’s *t*-test).

Given the sick phenotype of the *ΔypkA* null mutant and the absence of asexual spores, most of the phenotypic analyses were precluded. So, we constructed a conditional mutant in which the *ypkA* gene was placed under the control of the nitrate reductase (*niiA*) promoter. The strategy for mutant construction and the validation by Southern blot analysis are shown in Supplementary Figures S1C,D. The *niiA* promoter is induced by inorganic nitrate source (e. g., magnesium nitrate; MN) and repressed by organic nitrate source (e. g., ammonium tartrate; AT), as previously described (Hu et al., 2007). The mRNA abundance of the *ypkA* gene was determined in the *niiA::ypkA* strain by RT-qPCR, both in induction and repression conditions (Figure 2A). There was about sevenfold repression of *ypkA* in cells grown in AMM supplemented with AT, in comparison to cells transferred to AMM supplemented with MN. Similar results were obtained when *niiA::ypkA* strain was grown for 24 h in AMM supplemented with AT or MN without media transfer (data not shown).

Although the *niiA::ypkA* conditional mutant has a wild-type-like hyphal growth rate on AMM under inducing or repression conditions at 30, 37, or 45°C, the *niiA::ypkA* strain displayed a significant decrease in radial growth in comparison to the wild-type (Figures 2B,C). Under these conditions, *niiA::ypkA* strain grew approximately 30 and 50% less than the wild-type strain at 37° and 45°C, respectively. This finding was also confirmed by the lower germination kinetics observed for the *niiA::ypkA* strain at 37°C in liquid medium (Supplementary Figure S3). Abnormal hyphal morphology was also apparent when the *niiA::ypkA* strain was grown on liquid AMM + AT at 37°C for 12 h. Mutant hyphae are clearly dysmorphic with shorter length and truncated hyphal tips with knobby and abnormal branching (Figure 2D).

### YpkA Participates in the SL Biosynthesis Pathway and Interacts With MpkA and SakA MAP Kinases

The highly conserved signaling pathways involving MpkA and SakA MAP kinases are essential and have overlapping function to drive cell adaptation to cell wall and temperature stresses in *A. fumigatus* (Altwasser et al., 2015; Rocha et al., 2015; Bruder Nascimento et al., 2016), also influencing numerous other virulence traits (Jain et al., 2011; Valiante et al., 2015; Pereira Silva et al., 2017). *ypkA* encodes a protein kinase that plays undefined roles in cell wall integrity in *Aspergillus* species, although yeast Ypk1 and Ypk2 can activate the CWI MAPK Mpk1 (Schmelzle et al., 2002; Roelants et al., 2011). In addition, the function of these two MAP kinases in the SL biosynthesis control is unclear in *A. fumigatus*. Thus, to gain insights on how MpkA^MPK1^ and SakA^HOG1^ connect SL biosynthesis and CWI maintenance, we constructed the Δ*mpkA niiA::ypkA* and Δ*sakA niiA::ypkA* double mutants (Supplementary Figures S1E–H, respectively). Interestingly, *niiA::ypkA* strain grown in the presence of the cell wall stressors Congo Red (CR) or Calcofluor White (CFW) presented wild-type levels of susceptibility under *niiA*-repression condition (Supplementary Figure S4). The same results were obtained in the presence of caspofungin and azoles such as voriconazole and fluconazole (data not shown). In contrast, the Δ*mpkA niiA::ypkA* double mutant grows poorly in non-stressing control condition and is unviable in the presence of CR and CFW in comparison to the parental strains. These results suggest a conditional synthetic lethal interaction between *ypkA* and *mpkA* during cell wall stress, supporting the hypothesis that these two kinases are acting in parallel pathways to ensure cell survival (Supplementary Figure S4A) under cell wall damage. Since the sensitivity of Δ*sakA niiA::ypkA* was similar to that of the parental strains, no evidence for genetic interaction between *ypkA* and *sakA* was obtained under cell wall stress (Supplementary Figure S4B).

**Figure 3:**
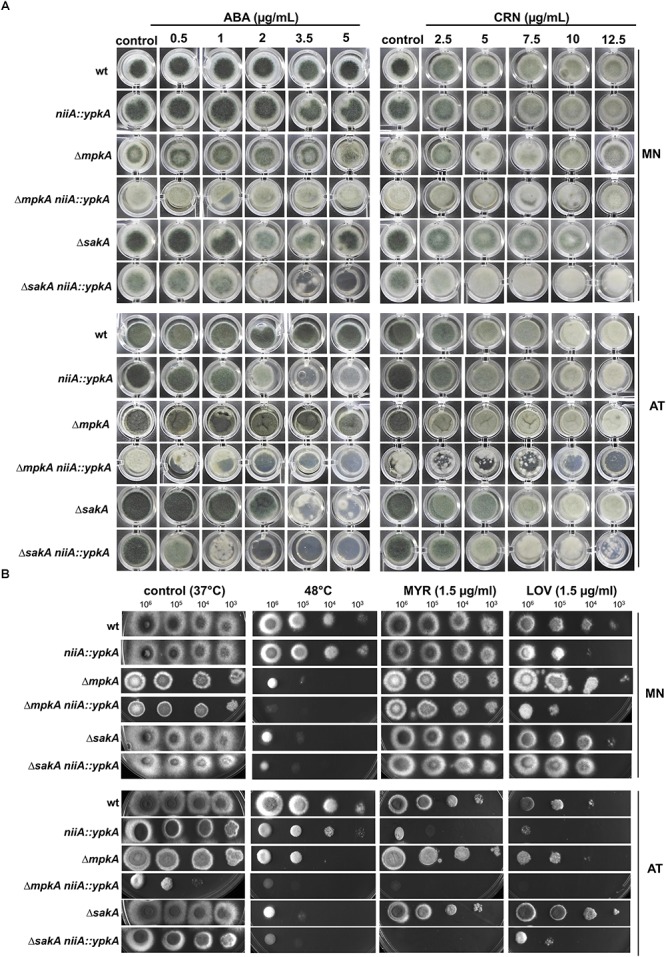
*ypkA* genetically interacts with the *mpkA* and *sakA* MAP kinases. **(A)** 1 × 10^4^ conidia of each strain were grown on 200 μl of solid AMM supplemented with magnesium nitrate (MN) or ammonium tartrate (AT) and the indicated concentrations of aureobasidin A (ABA) or cerulenin (CRN) in microtiter plates. **(B)** The indicated number of conidia of each strain was spotted on solid AMM supplemented with MN or AT containing the indicated concentration of myriocin (MYR) or lovastatin (LOV). Plates were incubated at 37 or 48°C for 48 h and photographed.

To determine if *ypkA* is connected with the CWI or HOG pathways during the inhibition of SL biosynthesis, we also performed genetic analysis using the double conditional mutants in the presence of molecules that impair the SL production at different steps of the biosynthetic pathway, such as (i) myriocin (MYR), an inhibitor of the serine palmitoyltransferase (SPT), which catalyzes the first and limiting step of SL biosynthesis (de Melo et al., 2013); (ii) aureobasidin A (ABA), an inhibitor of IPC synthase that abrogates the synthesis of IPCs, which are essential for fungal viability (Cheng et al., 2001); (iii) cerulenin (CRN) that inhibits fatty acid production and therefore can influence on the fatty acid amidation with LCB to produce SL; and (iv) lovastatin (LOV), which inhibits the rate-limiting enzyme HGM-CoA reductase and interferes in ergosterol biosynthesis, possible reflecting in the composition of lipid rafts (Chamilos et al., 2006). The *niiA::ypkA* mutant displayed increased sensitivity to ABA, MYR and LOV in comparison to the wild-type strain under repression conditions (Figures 3A,B). Noteworthy, the Δ*mpkA niiA::ypkA* double mutant but not Δ*sakA niiA::ypkA* displayed significant growth reduction in non-stressing conditions in the presence of AT (control) (Figure 3B). On the other hand, both double mutants were considerably more sensitive to ABA, CRN, MYR and heat stress in comparison to the parental strains (Figures 3A,B). Of note, among the single MAPK deletion strains, only Δ*sakA* displayed reduced tolerance to ABA, suggesting that activated SakA is required for SL synthesis and cell membrane homeostasis. Our analyses suggest that *ypkA* genetically interacts through parallel pathways with the two *A. fumigatus* MAPK MpkA and SakA exhibiting a conditional lethal phenotype upon the recovery from the SL biosynthesis inhibition and heat stress. Surprisingly, the Δ*sakA* and Δ*sakA niiA::ypkA* strains were more resistant to LOV than the parental strains, while Δ*mpkA niiA::ypkA* double mutant also exhibited conditional lethality in the presence of LOV (Figure 3B).

**Figure 4:**
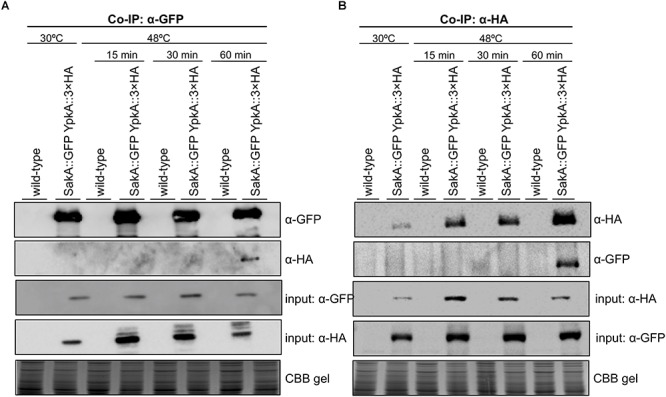
YpkA and SakA interact *in vivo* during heat shock stress. The wild-type and *sakA*::GFP *ypkA*::3xHA strains were used in the Co-IP assays. Strains were grown at 30°C (24 h) and subsequently exposed to heat shock at 48°C for the indicated times. **(A)** GFP-Trap resin was used to immunoprecipitate SakA::GFP. **(B)** Dynabeads Protein A were incubated with monoclonal α-HA antibody and used to immunoprecipitate YpkA::3xHA in reciprocal experiment. Co-immunoprecipitated proteins were investigated via Western blot analysis using α-HA and α-GFP antibodies. The Coomassie Brilliant Blue (CBB) stained gel was used as an additional loading sample control.

Based on the existing genetic interaction between *ypkA* and *mpkA* or *sakA* MAP kinases in the conditions above described, we sought to verify if these interactions occurred at the protein level by using co-immunoprecipitation (Co-IP) assays to further support the biological importance of the YpkA connection with the *A. fumigatus* MAP kinases. To test the physical interaction between SakA and YpkA, we used the double tagged *sakA*::GFP *ypkA*::3xHA generated by introducing a 3xHA tag at the C-terminus of YpkA in the background of the C-terminally GFP tagged SakA strain. Cellular extracts were obtained after submitting mycelia to heat shock at 48°C, since under this condition we observed conditional lethality for these two genes (Figure 3B) and also because heat shock is a known inducer of MpkA and SakA activation (Bruder Nascimento et al., 2016; Rocha et al., 2016). Co-IP assay was performed using GFP-trap beads with the wild-type and GFP-tagged SakA and 3xHA-tagged YpkA. The results indicate that there was an interaction between YpkA and SakA *in vivo* after 60 min of heat shock (Figure 4A). The immunoblotting analysis revealed that YpkA was only co-immunoprecipitated from the GFP-tagged SakA and 3xHA-tagged YpkA. To further confirm the specificity of this interaction, we performed reciprocal Co-IP assays using α-HA antibodies coupled to magnetic beads. Consistently, SakA is co-immunoprecipitated with YpkA after 60 min of heat shock (Figure 4B). Collectively, the results from the genetic analysis and the Co-IP ultimately suggest a role of SakA MAP kinase and the HOG pathway in YpkA activation upon heat shock and SL depletion. Noteworthy, physical interaction between yeast Ypk1^YpkA^ and Hog1^SakA^ homologs has not been described.

In contrast, we did not observe protein-protein interaction for YpkA and MpkA under the same experimental conditions, but using α-phospho p44/42 antibody that specifically recognizes *A. fumigatus* MpkA (Bruder Nascimento et al., 2016; Supplementary Figure S5).

**Figure 5:**
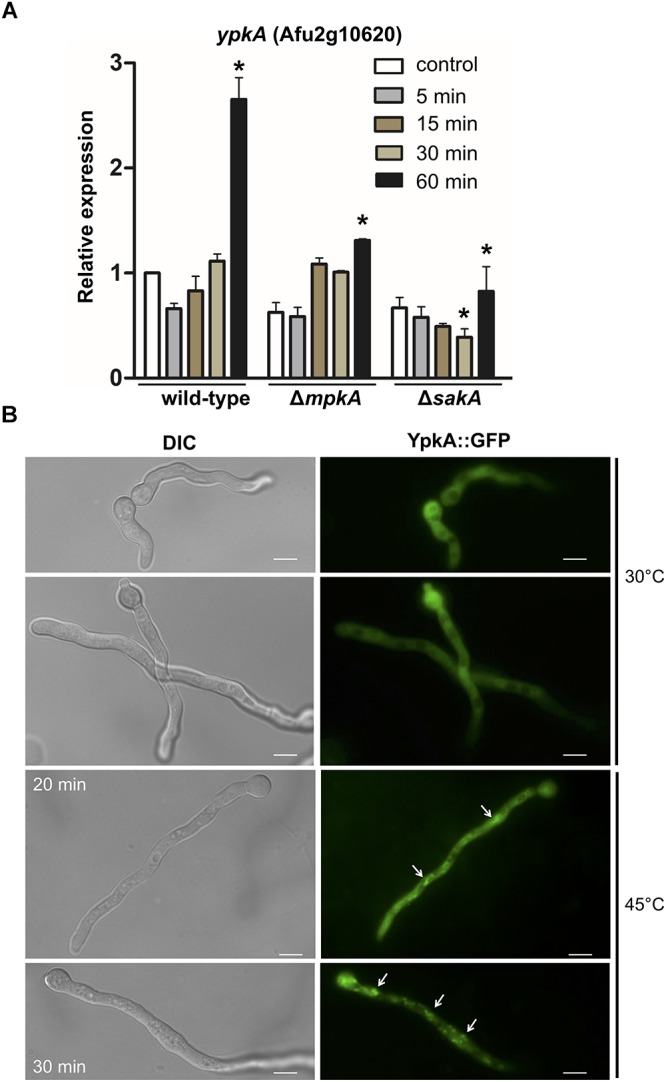
*ypkA* expression upon heat shock is perturbed in the absence of SakA and MpkA MAP kinases. **(A)** The wild-type, Δ*mpkA* and Δ*sakA* strains were grown at 30°C for 24 h in MM and subsequently transferred to fresh pre-warmed MM and grown for further 5, 15, 30, and 60 min at 48°C. mRNA abundance for *ypkA* gene was assessed by RT-qPCR and normalized to β-tubulin expression. Relative expression is shown by average ± SD (*n* = 3) of normalized mRNA abundance relative to wild-type at the same time point. ^∗^*p* ≤ 0.05 (one-way ANOVA). **(B)** YpkA localizes to the cytosol and reallocates in cytosolic aggregates after heat shock. The conidia of *ypkA*::GFP strain were inoculated in MM and incubated at 30°C for 16 h and transferred to 45°C for 20 or 30 min. The hyphae were directly inspected under the fluorescence microscope. Bars: 5 μm.

As a next step, we analyzed the expression of *ypkA* gene in the wild-type and in the Δ*mpkA* and Δ*sakA* deletion mutants during heat shock to seek for evidences if *ypkA* expression occurs in a *sakA* or *mpkA*-dependent manner. Here, we found that *ypkA* was significantly up-regulated in the wild-type strain after 60 min of heat shock (Figure 5A). In contrast, the inactivation of both *sakA* and *mpkA* significantly attenuated the *ypkA* transcriptional response (*p* ≤ 0.05), an observation that further supports the interaction between SakA and YpkA (Figure 4). Furthermore, we also investigated the phosphorylation profile of the MpkA and SakA MAP kinases in both the wild-type and *niiA::ypkA* strains in the presence of AT. The repression of *ypkA* led to an increased SakA phosphorylation after 30 and 60 min of heat shock exposure. Likewise, MpkA phosphorylation was also increased in the control condition and after 15 and 30 min of heat shock. Surprisingly, phosphorylated MpkA was not detected after 60 min of heat shock exposure in repressed *niiA::ypkA* strain (Supplementary Figure S6).

In fungi, plasma membrane also acts as a direct sensor of the outer environment and is one of the first cellular structures to detect ambient temperature fluctuations, changing considerably its fluidity and other physical and chemical properties (Shapiro and Cowen, 2012). The associated changes in the membrane fluidity to adapt temperature variations are achieved based on a complex balance between saturated and unsaturated fatty acids that constitute the plasma membrane (Leach and Cowen, 2014). We therefore examined the cellular distribution of YpkA by fluorescence microscopy in germlings exposed to heat shock. At 30°C, *ypkA*::GFP strain produced a strong and diffuse fluorescent signal along the germling throughout the cytoplasm. Interestingly, after 20–30 min of incubation at 45°C, cells displayed a noticeably sub cellular accumulation in the cytosol of the hyphae (Figure 5B), indicating that YpkA accumulates in specific cell regions during the heat shock.

### Loss of Function of YpkA Leads to an Increase in Ergosterol Content

**Figure 6:**
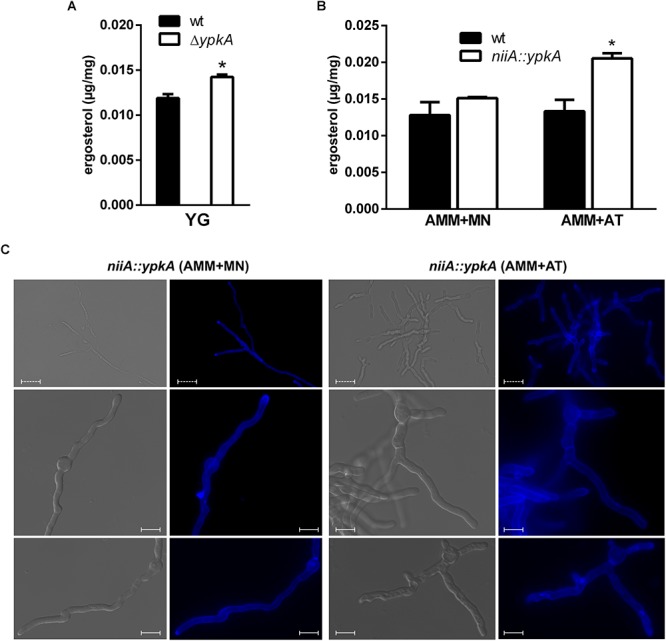
The content and cellular distributions of ergosterol are abnormal under *ypkA* loss of function. **(A)** 1 × 10^3^ conidia of wild-type strain and a cell amount of Δ*ypkA* mutant were inoculated in liquid YG culture and incubated at 37°C for 120 h before ergosterol extraction and quantification by HPLC (see section “Materials and Methods” for details). **(B)** 1 × 10^7^ conidia of both wild-type and *niiA::ypkA* strains were inoculated in liquid AMM supplemented with MN or AT and incubated at 37°C for 24 h before ergosterol extraction and quantification by HPLC. Experiments were performed in triplicate and the results are the mean ± SD. Results show the ergosterol concentration normalized by the mycelia dry weight. ^∗^Statistically significant (Student’s *t*-test; *p* ≤ 0.05). **(C)** 1 × 10^5^ conidia of *niiA::ypkA* strain were inoculated in liquid AMM supplemented with MN or AT and incubated at 37°C for 12 h. The germilings were stained with 25 μg/ml of filipin for 5 min, observed in a fluorescence microscope and photographed. Dashed bars = 20 μm. Solid bars = 10 μm.

To learn more about the effects of YpkA loss of function in *A. fumigatus*, we quantified the ergosterol content in the mutant strains. Ergosterol and SL are the components of membrane lipid rafts consisting of an aggregation of these molecules that mediates biosynthetic and endocytic processes by anchoring compounds to the plasma membrane (Alvarez et al., 2007). We verified that both, the deletion and the repression of *ypkA* in AMM + AT, resulted in a 25% increase in ergosterol (Figures 6A,B). This result suggests that cells may compensate the impairment in SL biosynthesis with an increased ergosterol accumulation to maintain cell membrane homeostasis in non-stressing conditions. Secondly, we analyzed whether the vegetative growth defects observed in both *ypkA* mutant strains were accompanied by defects in cell polarity and mislocalization of lipid rafts in germling cell membranes. For that purpose, we stained germilings of the *niiA::ypkA* strain with the fluorescent polyene filipin that binds sterols with high affinity. Although increased, the distribution of ergosterol was abnormal in the *niiA::ypkA* mutant under repressive conditions. When germlings were stained with filipin, the ergosterol accumulation was typically located at the hypal tip under inducing conditions (AMM + MN). However, fluorescence was misplaced in the *niiA::ypkA* mutant under repressive conditions (AMM + AT) and filipin staining was evenly distributed across the germilings without clear accumulation at the hyphal apex (Figure 6C). For the wild-type strain, ergosterol localization was observed at the hyphal apex regardless of the nitrogen source (data not shown).

### The SL Biosynthetic Pathway Is Affected by the Loss of *ypkA*

**Figure 7:**
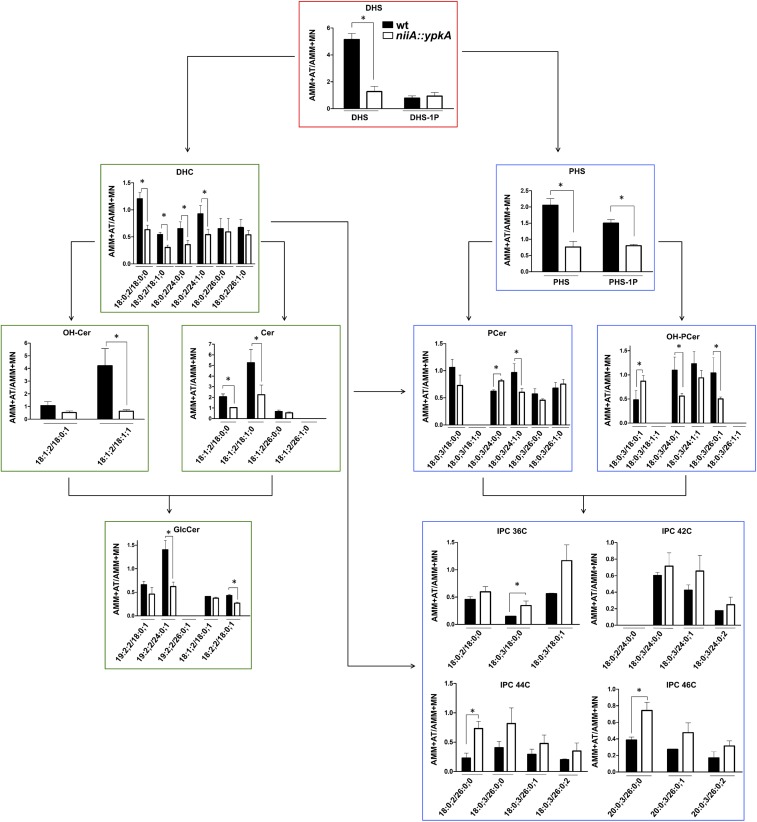
*ypkA* loss of function causes depletion of GlcCer and increase in IPCs abundance. The *niiA::ypkA* and wild-type strains were grown in AMM liquid medium supplemented with MN or AT for 24 h and subsequently subjected to sphingolipids extraction and quantification. Graphs show the ratio obtained by dividing SL levels (obtained as pmol/Pi) in AMM + AT and AMM + MN to normalize the inherent growth differences under these two nitrogen sources observed for the wild-type strain (see Figure 2). Graphs with red border indicate common intermediates of neutral and acidic GSL, while graphs with green and blue borders indicate exclusive intermediates of neutral or acidic GSL, respectively. Experiments were performed in independent triplicate and the results are the average ± SD. ^∗^Statistically significant by Student’s *t*-test (*p* ≤ 0.05). DHS, dihydrosphingosine; DHS-1P, dihydrosphingosine 1-phosphate; DHC, dihydroceramide; Cer, ceramide; GlcCer, glucosylceramide; OH-Cer, hydroxy-ceramide; PHS, phytosphingosine; PHS-1P, phytosphingosine 1-phosphate; PCer, phytoceramide; OH-PCer, hydroxy-phytoceramide; IPC, inositolphosphoryl ceramide. OH-Cer and OH-PCer refer to ceramides and phytoceramides, respectively, containing an extra-hydroxyl group at the fatty acid chain. All the SL abbreviations nomenclatures are expressed as Long-chain-base/Fatty-acyl. Long chain bases and fatty acyls are expressed as X:Y:Z (X, number of carbons; Y, number of C-C double bonds; Z, number of hydroxyl groups), according to (Grillitsch et al., 2014). The values of each SL obtained for each strain under the different conditions are shown in the Supplementary Table S4.

As an additional approach to understand the contribution of *ypkA* in the biosynthesis of both neutral (GlcCer) and acidic GSLs, the main SL intermediates downstream the enzyme serine palmitoyl transferase (SPT), including the IPCs, were further quantified in the *niiA::ypkA* conditional mutant using mass spectrometry (Figure 7). Briefly, acidic GSL differ from neutral GSL mainly because they contain an additional -OH group at C4 of the sphingoid base and lack C9-methylation and Δ4- and Δ8-unsaturations. Moreover, they contain a very long fatty acid instead of the C16-18 chain found in neutral GSL. For this reason, IPCs of 36, 42, 44, and 46 carbons with different number of hydroxyl groups were analyzed. Of note, the species shown in Figure 7 are the most abundant SL species observed in our analysis.

Overall, repression of *ypkA* in AMM + AT resulted in a significant decrease in GlcCer production, affecting almost all the SL classes measured (Figure 7). Unexpectedly, wild-type strain presents 4.8 times more DHS when cultured in AT medium instead of MN, on the other hand for the *niiA::ypkA* mutant almost no variation of DHS is observed (ratio = 1.2) when it was cultivated in medium containing AT or MN (Figure 7 and Supplementary Table S4). DHS is an upstream component of the SL pathway in addition to being the branching component in the SL biosynthesis, as it can generate two distinct pools of ceramide [dihydroceramide (DHC) and phytoceramide PCer)]. DHC and PCer are precursors for the formation of neutral or acidic GSL, respectively. Here, we observed lower levels of the most abundant GlcCer (19:2;2/24:0;1). Previous studies have shown several cellular defects being associated with lowered levels of GlcCer [reviewed in Del Poeta et al. (2014)]. Similarly, for acidic SL, the PCer precursor phytosphingosine (PHS) presented lower ratio in the *niiA::ypkA* mutant (Figure 7).

Surprisingly, the major exception lies in the inositolphosphorylceramide (IPC) class, in which intermediates displayed increased accumulation in the *niiA::ypkA* strain (i.e., high AT/MN ratio; Figure 7). Although lower ratio of the precursors phytosphingosine (PHS) and phytoceramide (PCer) or OH-PCer containing 24 and 26C were observed in the *niiA::ypkA* mutant, IPCs of 36, 44, and 46 carbons were significantly increased. Interestingly, an exception was seen for IPCs containing 42C. The IPC species detected are carrying either DHC, PCer or α-OH-PCer as a backbone with only saturated fatty acids. For some IPC species, such as 42:0;5, 44:0;5, 46:0;3, 46:0;4, and 46:0;5, the corresponding ceramide species were below detectable level.

**Figure 8:**
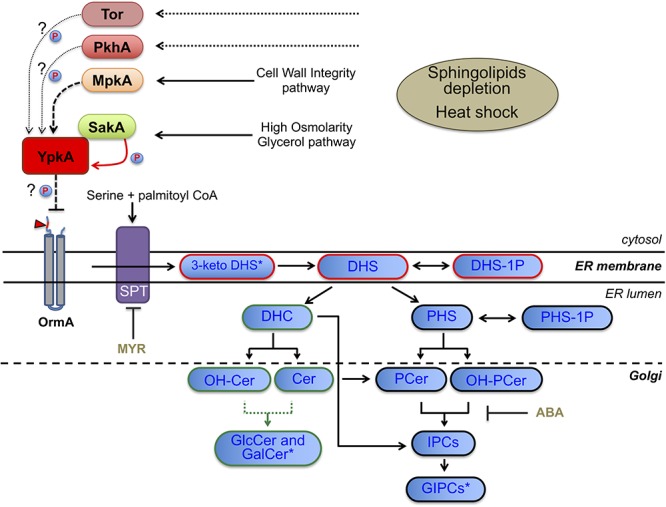
Scheme of the SL biosynthetic pathway in fungi, SL intermediates and proposed model for YpkA activation in *A. fumigatus*. Dashed arrow indicates genetic interaction between MpkA and YpkA while solid red arrow indicates genetic and physical interaction between SakA and YpkA. Dotted arrow indicates the predicted uninvestigated role of PkhA or Tor in phosphorylating YpkA. Arrowhead indicates the optimal consensus phosphoacceptor motif for yeast Ypk1 [-R-x-R-x-x-S/T] fully conserved and located at the N-terminal region (cytosolic side) of *A. fumigatus* OrmA (Afu4g13270), a predicted target of YpkA and SPT inhibitor (Roelants et al., 2011; Frohlich et al., 2016). SPT, serine palmitoyl transferase; MYR, myriocin; ABA, aureobasidin (blocks the synthesis of IPC from either PCer or OH-PCer); P, kinase activity over YpkA. For SL intermediates abbreviations see Figure 7. Blue boxes with red border indicate common intermediates of neutral and acidic GSL, while blue boxes with green and black borders indicate exclusive intermediates of neutral or acidic GSL, respectively. Green dotted line represents the two enzymes necessary for GlcCer and GalCer production, i.e., Sld8 (which desaturates Cer and OH-Cer in position 8 of the sphingosine backbone) and Smt1 (which methylates the Δ8 Cer and Δ8-OH-Cer in position 9 of the sphingosine backbone). ^∗^Not measured (Figure 7): 3-keto DHS, 3-keto dihydrosphingosine; GalCer, galactosylceramide; GIPCs, glycosylinositol phosphorylceramide.

Altogether, our results suggest that the neutral branch of the SL biosynthetic pathway is more affected by the YpkA loss of function resulting in lower ratios of GlcCer than the acidic branch, which forms acidic glycosphingolipids, including the IPCs (Figure 8).

## Discussion

Unlike other subcellular membranes, plasma membrane is enriched with SL and sterols which are organized in lipid microdomains (Alvarez et al., 2007). It has been suggested that SL unbalance interferes in fungal virulence (Rella et al., 2016), but the signaling pathways that coordinated the *de novo* synthesis of SL are poorly understood in *A. fumigatus*. Here we characterized the yeast *YPK1/YPK2* homolog in *A. fumigatus* (*ypkA*) that encodes a kinase acting on the SL-mediated signaling pathway (Sun et al., 2000; Roelants et al., 2010) and analyzed its function in SL biosynthesis, as well as its connection with the CWI and HOG pathways.

Previously we characterized the AGC kinase PkcA (Rocha et al., 2015, 2016) and learned that another coding sequence in the *A. fumigatus* genome shares high homology with the PkcA catalytic site, thus displaying full conservation of residues involved in phosphoryl transfer (Supplementary Figure S2). This sequence was further validated as the single yeast Ypk1/2 homolog in *A. fumigatus*. Similarly to *A. nidulans* (Colabardini et al., 2013), a null *ypkA* mutant displayed a drastically sick phenotype and complete absence of conidiation (Figure 1). Additional evaluation of the *niiA::ypkA* conditional mutant also demonstrated that this kinase plays a pivotal role in fungal growth and vegetative proliferation (Figure 2 and Supplementary Figure S3) even though this conditional mutant does not present the same sick phenotype displayed by the null mutant (Figures 1, 2). A possible explanation is the limitation of the nitrogen-regulated *niiA* promoter to completely suppress the *ypkA* transcription, thus leaking during repression condition. This has also been reported previously in *A. fumigatus* (Hu et al., 2007; Li et al., 2011).

In yeast, single *YPK1* or *YPK2* deletion is viable while the double mutant is lethal (Roelants et al., 2002). Despite the terminal phenotype, propagation of Δ*ypkA* aconidial colonies was feasible, thus suggesting that under non-stressing conditions, biochemical modification of cell wall or cell membrane, especially encompassing ergosterol and SL content, sustain cell survival. Accordingly, we describe that ergosterol accumulation is increased in both *ypkA* loss of function mutants, suggesting that this small increase in ergosterol may be important for cell adaptation in unchallenged cells when SL synthesis is impaired (Figure 6). Importantly, *niiA::ypkA* conditional mutant under repression exhibited increased sensitivity to lovastatin and altered localization of ergosterol-rich membrane domains suggesting that similar to yeasts, YpkA may act as a sensor of ergosterol and SL levels to promote cell membrane homeostasis. Indeed, low ergosterol concentration stimulates Ypk1 activity (Li et al., 2010; Roelants et al., 2011). Consequently, when both SL and ergosterol biosynthesis were disturbed, cells lose viability (Figure 3B). Intriguingly, we observed that SakA has a possible negative role in ergosterol production as Δ*sakA* and Δ*sakA niiA::ypkA* strains displayed increased tolerance to lovastatin. It remains to be determined how SakA controls ergosterol biosynthesis pathway and whether SakA interacts with proteins involved in ergosterol production.

As expected, the *ypkA* loss of function mutant showed increased susceptibility to MYR, ABA and temperature stress. Our results suggest that among other functions, *ypkA* alleviates negative regulation of SPT, thus up-regulating LCB production (Figures 2, 3, 7). Apparently, the *de novo* biosynthesis of neutral GSL such as DHC, Cer, OH-Cer and GlcCer seems to be required to support membrane integrity in the absence of the *ypkA*, since these intermediates were less abundant upon *ypkA* repression. In accordance, the SL precursors DHS and PHS are most affected in the mutant (Figure 7), further supporting the role of *ypkA* in the SL biosynthesis. Importantly, *S. cerevisiae* does not present Gcs1, Sld8, or Smt1 enzymes (Figure 8). Consequently, *S. cerevisiae* cannot produce GlcCer and the Cer species necessary to produced GlcCer (Del Poeta et al., 2014). In contrast, *A. fumigatus* encodes these two enzymes and therefore synthetizes GlcCer, which are necessary for fungal virulence in fungal pathogens. Here, GlcCer is decreased in *niiA::ypkA* mutant (Figure 7), thus suggesting that YpkA is required to produce this specific SL, being possible important to support *A. fumigatus* virulence. It is interesting that for certain IPC species (42:0;5, 44:0;5, 46:0;3, 46:0;4, and 46:0;5) we could not detect the direct ceramide substrate, suggesting that as soon as these substrates are produced, they are rapidly utilized by the Ipc1 synthase to make the corresponding IPCs species. Also, we could not detect any IPCs carrying unsaturated fatty acid, suggesting that fatty acid unsaturation may be not compatible with the production of IPCs species in *A. fumigatus* regulated by YpkA, as the level of unsaturation will destabilize the membrane and decrease the melting temperature.

Temperature is a determining factor for the maintenance of plasma membrane properties (Shapiro and Cowen, 2012). Here *ypkA* mRNA abundance was increased during heat shock (Figure 5A). Accordingly, we observed that at 30°C YpkA is dispersed throughout the cytoplasm of the hyphae, while it reallocated in aggregates within the cytoplasm upon heat shock (Figure 5B). While in *S. cerevisiae* Ypk1 is located exclusively in the cytosol and Ypk2 is found in nucleus (Roelants et al., 2002), we failed to identify any nuclear localization of *A. fumigatus* YpkA under the conditions we employed, suggesting that the cytosolic targets of this enzyme, such as those responsible for the synthesis of sphingolipids, are more important for normal growth in *A. fumigatus*.

Eukaryotes possess highly conserved MAPK cascades, which regulate cell physiology against environmental changes, such as heat shock, disruption of cell wall integrity or osmotic stresses (Bruder Nascimento et al., 2016; Hagiwara et al., 2016). In *A. fumigatus*, these cascades culminate in the activation of four downstream MAP kinases to in turn activate target proteins causing adaptive cellular responses (Hagiwara et al., 2016). In *A. fumigatus*, MpkA and SakA are the MAPKs of CWI and HOG pathways, respectively (Xue et al., 2004; Valiante et al., 2008; Bruder Nascimento et al., 2016; Rocha et al., 2016). Previous studies have pointed out a connection between these proteins and the SL biosynthesis in *S. cerevisiae* (Roelants et al., 2002; Schmelzle et al., 2002; Tanigawa et al., 2012; Yamaguchi et al., 2017), and our results unveil that these interactions also occur in *A. fumigatus*. Both *mpkA* and *sakA* genetically interact with *ypkA* though parallel pathways, in view of the increased sensitivity of the double mutants to ABA, CRN, MYR and heat stress (Figure 3). Consistently, phosphorylation of both SakA and MpkA were increased in repressed *niiA::ypkA* strain (Supplementary Figure S6). Furthermore, in the absence of the *mpkA* and *sakA* genes, there is lower expression of *ypkA* (Figure 5A). Collectively, the results suggest that these MAPK are important for signaling the initial steps of SL biosynthesis. Likewise, absence of Mpk1 was lethal in *S. cerevisiae* YPK1/2 mutants (Roelants et al., 2002). Despite the large evidences for genetic interaction observed between these two MAPK and *ypkA*, we demonstrate here that only SakA physically interacts with YpkA under the conditions tested (Figure 4). Interestingly, this interaction is not described for yeast counterparts. Frohlich et al. (2016) analyzed the global cellular response to SL depletion caused by MYR in yeast though phosphoproteomic approach. Among the up-regulated phosphorylation sites, the likelihood of a proline at the +1 position relative to the S/T is significantly decreased, indicating that MAP kinases likely do not play a major role in mediating the response to MYR in terms of the downstream metabolic enzymes involved in SL biosynthesis (Frohlich et al., 2016). In contrast, our results suggest that the upstream activation of SL signaling requires the MAPK SakA. It is known that yeast 3-phosphoinositide-dependent kinase 1 orthologs Pkh1/2 (Roelants et al., 2002) and the target of rapamycin complex 2 (Torc2) (Niles et al., 2012; Muir et al., 2014) are responsible for Ypk1 phosphorylation and a similar scenario may occur in *A. fumigatus*. Although the participation of the *A. fumigatus* single homologs PkhA and Tor (Baldin et al., 2015) in YpkA phosphorylation was not investigated, deletion of *pkhA* (Afu3g12670) phenocopies Δ*ypkA* strain (data not shown). Comparable result was previously observed in *A. nidulans* (Colabardini et al., 2013), supporting the assumption that YpkA is also a target of PkhA. Currently, no information about the role of Tor kinase in SL biosynthesis is available. Considering that the interaction of YpkA with the MAPK SakA reported here is possible a late event in the SL pathway activation, occurring 60 min after heat shock, we suggest that prior the YpkA activation via interaction with SakA, YpkA can be activated earlier by other activating kinases such as Tor and/or PkhA. SakA is known to be quickly activated in response to different cell stresses such as osmotic and cell wall stress (Du et al., 2006; Alves de Castro et al., 2016). Here, the activation of SakA under temperature stress was more evident only when SL biosynthesis is disturbed (Supplementary Figure S6), suggesting that this MAPK is indirectly required to maintain cell membrane homeostasis. Regardless which putative upstream kinases may activate YpkA (Figure 8), the downstream YpkA targets are unknown in *A. fumigatus*. One of the Ypk1 targets in yeast is the Orm family proteins, which are conserved integral membrane proteins resident in the endoplasmic reticulum and inhibitors of SPT activity. All seven amino acid residues of the consensus phosphoacceptor motif (RRRRSSS) located on the N-terminus cytoplasmic region of yeast Orm1 and Orm2, which are phosphorylated by Ypk1 (Roelants et al., 2011), are fully conserved in the single *A. fumigatus* OrmA ortholog (Figure 8 and data not shown). This again suggests a function of YpkA as an inhibitor of OrmA and an activator of SL production.

It has been demonstrated that some SL metabolism enzymes are also Ypk1 direct targets (Muir et al., 2014). Among them, *LAG1*/*LAC1* are ceramide synthases which catalyze the reaction whereby DHS is amide-linked to a C26 fatty acid to yield DHC and Cer (reviewed in Dickson, 2008; Fernandes et al., 2018). As aforementioned, we observed decreased levels of DHC and Cer (neutral GSL) while most of IPCs (acidic GSL) were produced at normal or slightly increased levels in the *niiA::ypkA* mutant (Figures 7, 8). In *A. nidulans*, *barA*^LAG1^ generates the ceramide pool involved in the neutral GSL synthesis (Fernandes et al., 2018) and genetically interacts with *ypkA* (Colabardini et al., 2013). Our data reinforces the connection between *ypkA* and Cer synthesis in *A. fumigatus* and suggest that a physical interaction between YpkA and BarA may also occur.

In summary, this is the first report indicating how *A. fumigatus* SL biosynthesis is affected when YpkA-mediated signaling cascade is compromised. It remains to be determined the specific conditions which SakA (and possibly PkhA or Tor kinases) are required for YpkA activation. Also, due to the poor growth and lack of conidiation, the importance of *ypkA* for virulence in animal models remains to be elucidated.

## Author Contributions

IM planned and designed the research. JF, NG, MR, MM, TC, TF, and IM performed the research. IM, MvZK, TF, AdC, and MDP contributed to reagents and analytic tools. IM, JF, MM, TF, and MDP analyzed and validated the data. IM and JF wrote the original draft of the manuscript. All authors contributed to manuscript revision, read, and approved the submitted version.

## Conflict of Interest Statement

MDP is a Co-founder and Chief Scientific Officer (CSO) of MicroRid Technologies Inc. The remaining authors declare that the research was conducted in the absence of any commercial or financial relationships that could be construed as a potential conflict of interest.

## Footnotes

1 http://www.aspgd.org
