# Supplementary material for: The AGC Kinase YpkA Regulates Sphingolipids Biosynthesis and Physically Interacts With SakA MAP Kinase in Aspergillus fumigatus
Source: Front Microbiol. 2019 Jan 14;9:3347. doi: 10.3389/fmicb.2018.03347 (PMC6339957; doi:10.3389/fmicb.2018.03347)
Supplement: Supplementary file 1 [file Data_Sheet_1.pdf]

Supplementary Material

The AGC kinase YpkA regulates sphingolipids biosynthesis and physically interacts with SakA MAP kinase in *Aspergillus fumigatus*

João Henrique Tadini Marilhano Fabri[1#], Naiane Lima Godoy[1#], Marina Campos Rocha[1], Mansa Munshi[4], Tiago Alexandre Cocio[1§], Marcia Regina von Zeska Kress[2], Taicia Pacheco Fill[3], Anderson Ferreira da Cunha[1], Maurizio Del Poeta[4,5,6,7], Iran Malavazi[1*]

[1]Departamento de Genética e Evolução, Centro de Ciências Biológicas e da Saúde, Universidade Federal de São Carlos, São Carlos, SP, Brazil.

[2]Departamento de Análises Clínicas Toxicológicas e Bromatológicas, Faculdade de Ciências Farmacêuticas de Ribeirão Preto, Universidade de São Paulo, Ribeirão Preto, São Paulo, Brazil.

[3]Instituto de Química, Universidade Estadual de Campinas, Campinas, São Paulo, Brazil.

[4]Department of Molecular Genetics and Microbiology, Stony Brook University, Stony Brook, NY, USA.

[5]Division of Infectious Diseases, School of Medicine, Stony Brook University, Stony Brook, New York, USA

[6]Institute of Chemical Biology and Drug Discovery, Stony Brook University, Stony Brook, NY, USA

[7]Veterans Administration Medical Center, Northport, NY USA.

§ Current Address: Faculdade de Medicina de Ribeirão Preto - FMRP/USP, Hospital das Clínicas de Ribeirão Preto

# Both authors equally contributed to this work

*** Correspondence:** Iran Malavazi: imalavazi@ufscar.br

1 Supplementary Material and Methods

Sequence analysis

The following protein sequences: YpkA (PkcB) from *A. nidulans* (AN5973), *S. cerevisiae* Ypk1 and Ypk2 (YKL126W and YMR104C, respectively), SGRK2 from *Homo sapiens* (GenBank: AAV38901.1) as well as the *A. fumigatus* PkcA (Afu5g11970) were used to identify similar sequences at the AspGD (http://www.aspergillusgenome.org). The selected sequences were downloaded and used in alignment

and scoring protocols using the Clustal Ω alignment tool (Sievers et al., 2011) available at <http://www.ebi.ac.uk/Tools/msa/clustalo/>. The position of the domains was identified through analysis with the Protein Data Bank (PDB) and the SIB Bioinformatics Resource Portal (<http://expasy.org>) and based on supporting literature describing AGC kinase structure (Casamayor et al., 1999; Herrmann et al., 2006; Sobko, 2006; Steinberg, 2008). The graphical representations were performed using the Jalview (2.10.1) software (Waterhouse et al., 2009) available at <http://www.jalview.org>.

Deletion of the *ypkA* gene and construction of the *niiA::ypkA*, *ypkA::*GFP and *ypkA::*3×HA strains

In brief, two fragments encompassing the *ypkA* (Afu2g10620) gene were PCR-amplified from genomic DNA of the CEA17 strain according to Figure S1A. The primers used are listed in Table S2. The 5’ and 3’ *ypkA* flanking sections contained a short sequence that was homologous to the multiple cloning site of the pRS426 plasmid (the small letters indicated in Table S2). The *pyrG* gene that was inserted into the gene replacement cassette was amplified from pCDA21 plasmid (Chaveroche et al., 2000) and used as a prototrophy marker. Deletion cassette was generated by transforming the three independent fragments along with the *Bam*HI-*Eco*RI-cut pRS426 into the *S. cerevisiae* FGSC 9721 strain as described in Malavazi and Goldman (2012). Genomic DNA that was extracted from the *S. cerevisiae* transformant cells was used to transform *Escherichia coli* chemocompetent DH5α cells to rescue the recombined pRS426 plasmid harboring the gene replacement cassette. The isolated plasmid was used as a template to PCR-amplify the cassette with the outermost primers (5F and 3R) indicated in Figure S1A. All the PCR amplifications were performed using Phusion Flash High-Fidelity DNA Polymerase (Thermo Scientific). The gene replacement cassette was transformed into protoplasts of the *A. fumigatus* ΔKU80 pyrG1 according to previously described procedures (Malavazi and Goldman, 2012). Transformants were carefully tested by PCR with primers ypkA 500 ups and pyrG REV to confirm the *ypkA* deletion (Figure S1B).

For *niiA::ypkA* conditional mutant construction, the cassette containing the *pyrG* gene and the nitrate reductase promoter (*niiA*) was PCR-amplified from the *A. nidulans niiA::ypkA* strain (a gift from Dr. Gustavo Henrique Goldman, FCFRP-USP, Brazil) and cloned in-frame with the *ypkA* genomic sequence according to Figure S1C. The *A. fumigatus niiA::ypkA* cassette was recombined *in vivo* in *S. cerevisiae* and amplified to be transformed into the *A. fumigatus* wild-type strain. Transformants were validated by Southern blot analysis, using the 5’ flanking region as probe (Figure S1D).

To generate translational fusion of YpkA with green fluorescent protein (GFP), a substitution cassette was constructed in which an *ypkA* genomic sequence without stop codon was cloned in-frame with the GFP gene in a C-terminal fusion (Figure S1I). Part of *ypkA* ORF was amplified by primers ypkA 1400 FW and ypkA ORF REV. A four-residue linker consisting of Gly-Thr-Arg-Gly was inserted between the C-terminus of the *ypkA* gene and the start codon of GFP, as described previously (Teepe et al., 2007). The GFP gene (726 bp) was PCR-amplified from the pMCB17apx (Fernandez-Abalos et al., 1998) plasmid by using the primers Spacer GFP FW and pyrG REV. The *pyrG* gene was also used as a marker for prototrophy. The amplification of the *ypkA* 3’ UTR region was performed with the same primers that were used in the construction of the deletion cassette, *i.e.*, Afu2g10620 3F and Afu2g10620 3R (Table S2). The *ypkA::*GFP cassette was recombined *in vivo* in *S. cerevisiae* and transformed into the *A. fumigatus* wild-type strain. Transformants were carefully tested by PCR with primers ypkA 500 ups and GFP 200 REV to confirm the *ypkA* locus replacement (Figure S1J) and ypkA 1400 FW and Afu2g10620 3R (Figure S1K) to discriminate the *ypkA* fusion from the wild-type *ypkA* locus.

The same methodology was used to construct the *ypkA::*3×HA strain, in which the 3×HA tag was amplified from the pUC 3×HA *prtA* plasmid (kindly provided by Dr. Gustavo Henrique Goldman, FCFRP-USP, Brazil), a derivative of pOB430 (kindly provided by Dr. Ozgur Bayram), by using the primers Linker 3×HA pyrG FW pOB430 and prtA REV (Figure S1L). The pyrithiamine-resistant transformants were selected and tested by PCR with primers ypkA 1400 FW and Afu2g10620 3R (Figure S1M) to discriminate the *ypkA* fusion from the wild-type *ypkA* locus and ypkA 500 ups and prtA REV to confirm the *ypkA* locus replacement (Figure S1N).

2 Supplementary Tables

Supplementary Table 1. *A. fumigatus* strains used in this study.

Name	Genotype	Reference
ΔKU80 pyrG1 (FGSC A1160[a])	Δ*akuB; pyrG- MAT1-1*	(da Silva Ferreira et al., 2006)
Δ*ypkA*	Δ*ypkA::pyrG;* Δ*akuB*	This study
niiA::ypkA	*niiA::ypkA*; Δ*akuB*	This study
*ypkA::*GFP	*ypkA::*GFP*;* Δ*akuB*	This study
*ypkA::*3×HA	*ypkA::*3×HA*;* Δ*akuB*	This study
*sakA::*GFP	*sakA::*GFP*;* Δ*akuB*	(Bruder Nascimento et al., 2016)
Δ*sakA*	Δ*sakA::hph;* Δ*akuB*	(Altwasser et al., 2015)
*sakA::*GFP *ypkA::*3×HA	*sakA::*GFP; *ypkA::*3×HA*;* Δ*akuB*	This study
Δ*mpkA*	Δ*mpkA::ptrA;* Δ*akuB*	(Valiante et al., 2009)
Δ*mpkA niiA::ypkA*	Δ*mpkA::ptrA; niiA::ypkA;* Δ*akuB*	This study
Δ*sakA niiA::ypkA*	Δ*sakA::hph*; *niiA::ypkA;* Δ*akuB*	This study
Δ*pkhA*	Δ*pkhA::pyrG;* Δ*akuB*	This study

[a]FGSC: Fungal Genetics Stock Center (http://fgsc.net)

Supplementary Table 2. Primers used in this study for construction of mutant strains.

Primer name*	Sequence (5’→3’)
Afu2g10620 5 F	gtaacgccagggttttcccagtcacgacgGAGTTGTAGATGTCGACCACG
Afu2g10620 5R	gcctcctctcagacagaattcCAGAAGATAGACGGATGTCCAGAAC
pyrG FW	GGAATTCTGTCTGAGAGGAGGC
pyrG REV	GATATCGAATTCGCCTCAAAC
Afu2g10620 3F	gtttgaggcgaattcgatatcTTAGCTCTTGAGTGTTGCTAACAG
Afu2g10620 3R	gcggataacaatttcacacaggaaacagcCTCAAGGATGTTCCTCGG
ypkA 500 ups	CGTTACTGGCAAAAGCTCG
ypkA 5R niiA	aggttcattggagctcgtatttttccctgcAGAAGATAGACGGATGTCCAGAAC
pyrG k7 niiA	GCAGGGAAAAATACGAGCTCC
niiA k7 niiA	CGTGACGAAGTCTCAACGCC
ypkA niiA	ggtcaatactggcgttgagacttcgtcacgATGTCGTGGAAGCTCACTAAAA
ypkA 1884 REV SC	gcggataacaatttcacacaggaaacagcAAAAGGTCACGAGCGGC
ypkA 1400 FW	gtaacgccagggttttcccagtcacgacgTTGAAGAAGCTCAACCATATGT
ypkA ORF REV	agttcttctcctttactcattccccgtgttccCTCAGGAATGCTGCCGAAGGAT
Spacer GFP FW	**GGAACACGGGGA**ATGAGTAAAGGAGAAGAACTTTTCA
GFP 200 REV	TGATCTGGGTATCTTGAAAAGC
ypkA ORF REV HA	accaccgctaccacctccCTCAGGAATGCTGCCGAAGGAT
Linker 3×HA pyrG FW	GGAGGTGGTAGCGGTGGT
pOB430	
prtA REV	CTATCATGGGGTGACGATGAGCCG
prtA ypkA 3 FW	cggctcatcgtcaccccatgatagTTAGCTCTTGAGTGTTGCTAACAG
MpkA 5’ FW[§]	CTCATTCCTTGTTCTGATGCG
MpkA 3’ REV[§]	GACTGTCGCAGAAATCCGCTT
mpkA 600 ups	GAGCCCTGACTTCACTGCA
SakA yes FOR	GTCTGTCTAAGGCAATATCG
SakA yes REV	CTATGGAGTGATCCCCGTCG’
SakA 500 ups	AGAACAGGAGCAGGACTGGT
sakA pET15b part 2 FW	GCGGCAGCCATATGCTCGAGATGATTTACTTTGTCACAGAGC
sakA pET15b part 3 REV	TCGGGCTTTGTTAGCAGCCGCTATAGAAGATCGACCGC

Small letters indicate homology to the pRS426 flanking sequence
Small underlined letters indicate homology to a fragment in the cassette
Bold letters indicate the Gly-Thr-Arg-Gly linker separating *ypkA* C-terminus and GFP start codon.
* For primers location refer to Figure S1.
§ Sequence from Valiante et al. (2009).

Supplementary Table 3. Real-time qPCR primers used in this study

Gene	Systematic name	Primer name	Sequence
ypkA	Afu2g10620	ypkA 1133 FW	5’-AGTTGTTCCACCACCTCCAG-3’
		ypkA 1260 REV	5’-GGGCTTCAAATCACGGTAGA-3’
tubA	Afu1g10910	tubA FW	5’-TTCCCAACAACATCCAGACC-3’
		tubA REV	5’-CGACGGAACATAGCAGTGAA-3’
mpkA	Afu4g13720	mpkA FW	5’-GGCCATCAAGAAGGTTACCA-3’
		mpkA REV	5’-TGAAATTGTCTGGTCGTGGA-3’

3 Supplementary Figures

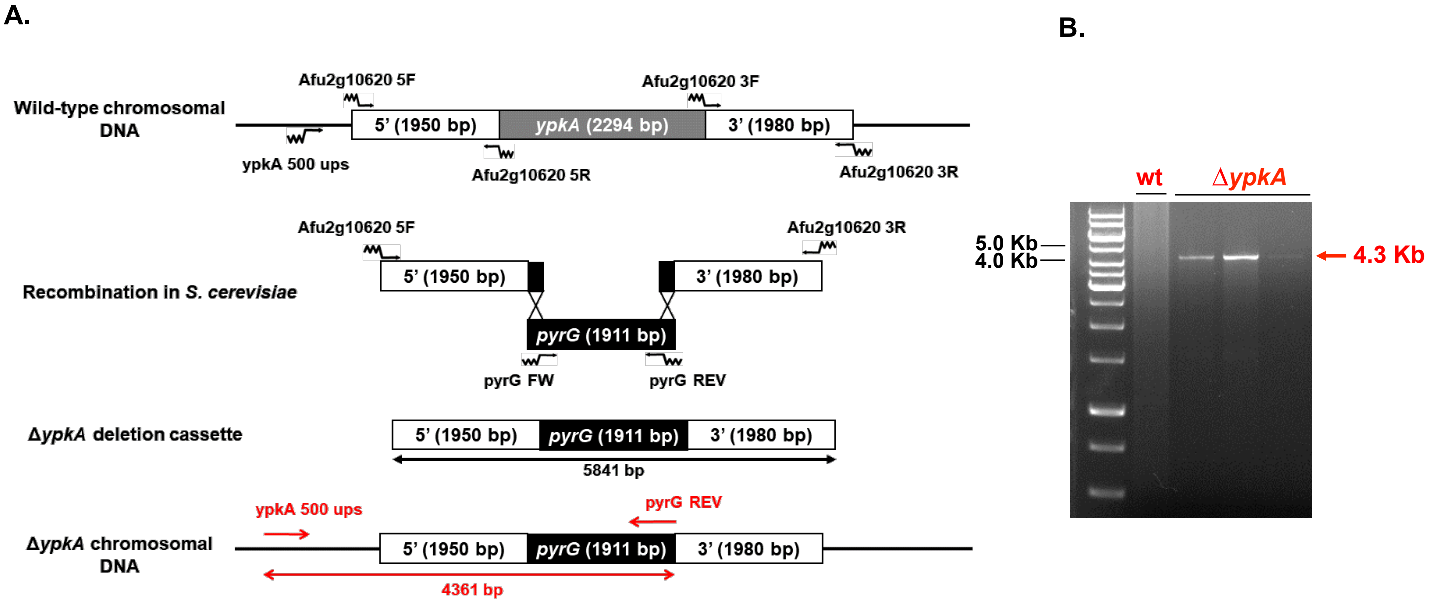

Supplementary Figure 1. Generation of the Δ*ypkA* mutant, *niiA::ypkA, ypkA::*GFP*, ypkA::*3×HA and double mutant strains. **(A)** Gene replacement strategy for the *ypkA* gene. The *pyrG* gene was used as a selection marker. The primer names and binding sites are indicated by arrows (primer sequences are described in Table S1). The deletion cassette was constructed by *in vivo* recombination in *S. cerevisiae*. **(B)** Diagnostic PCR to evaluate the *ypkA* locus after gene replacement using a primer located 500 bp upstream of the deletion cassette, as shown by red lines and red letters. **(C)** Gene replacement strategy for *niiA::ypkA* conditional mutant construction. The nitrate reductase promoter (*niiA*) was cloned in-frame with the *ypkA* genomic sequence and the recombination was performed *in vivo* in *S. cerevisiae.* **(D)** Southern blot analysis for the conditional mutant. *Apa*I-digested genomic DNA with a probe that binds specifically to the *ypkA* 5'-region indicated the predicted 4.1 Kb band in the *niiA::ypkA* mutant, as shown by blue lines and blue letters. **(E-F)** PCR for the validation of the Δ*mpkA niiA::ypkA* double mutant using the mpkA 600 ups and mpkA 3' REV primers **(E)**, and mpkA FW and mpkA REV primers **(F)**, with the objective of verifying the absence of the ORF amplification in the double mutant. **(G-H)** PCR for the validation of the Δ*sakA niiA::ypkA* double mutant using the primers sakA 500 ups and SakA yes REV **(G)** and sakA pet 15b parte 2 FW and sakA pet 15b parte 3 REV **(H)**. **(I)** Gene replacement strategy for *ypkA::*GFP strain construction. The *ypkA* genomic sequence with no stop codon was cloned in-frame with the green fluorescent protein (GFP) gene in a C-terminal fusion separated by a Gly-Thr-Arg-Gly linker. The *pyrG* gene was also used as a prototrophy marker. *S. cerevisiae in vivo* recombination assay was performed and the cassette was transformed into the *A. fumigatus* wild-type strain. **(J-K)** Diagnostic PCRs to evaluate the *ypkA* locus after gene replacement using the primers shown in orange **(J)** and in purple **(K)**. The correct amplifications confirm the achievement of the *ypkA::*GFP mutant. **(L)** Gene replacement strategy for *ypkA::*3×HA strain construction. The *ypkA* genomic sequence with no stop codon was cloned in-frame with the 3×HA tag in a C-terminal fusion. The phyrithiamine resistance gene was used as selection marker. *S. cerevisiae in vivo* recombination assay was performed and the cassette was transformed into the *A. fumigatus* wild-type strain. **(M-N)** Diagnostic PCRs to evaluate the *ypkA* locus after gene replacement using the primers shown in blue **(M)** and in red **(N)**. The correct amplifications confirm the achievement of the *ypkA::*3×HA mutant. **(O)** PCR for the validation of the *sakA*::GFP *ypkA*::3×HA double mutant using the ypkA 500 ups and prtA REV primers. **(P-Q)** Western blot for the validation of the *sakA*::GFP *ypkA*::3×HA double mutant using α-HA **(P)** and α-GFP **(Q)** antibodies.

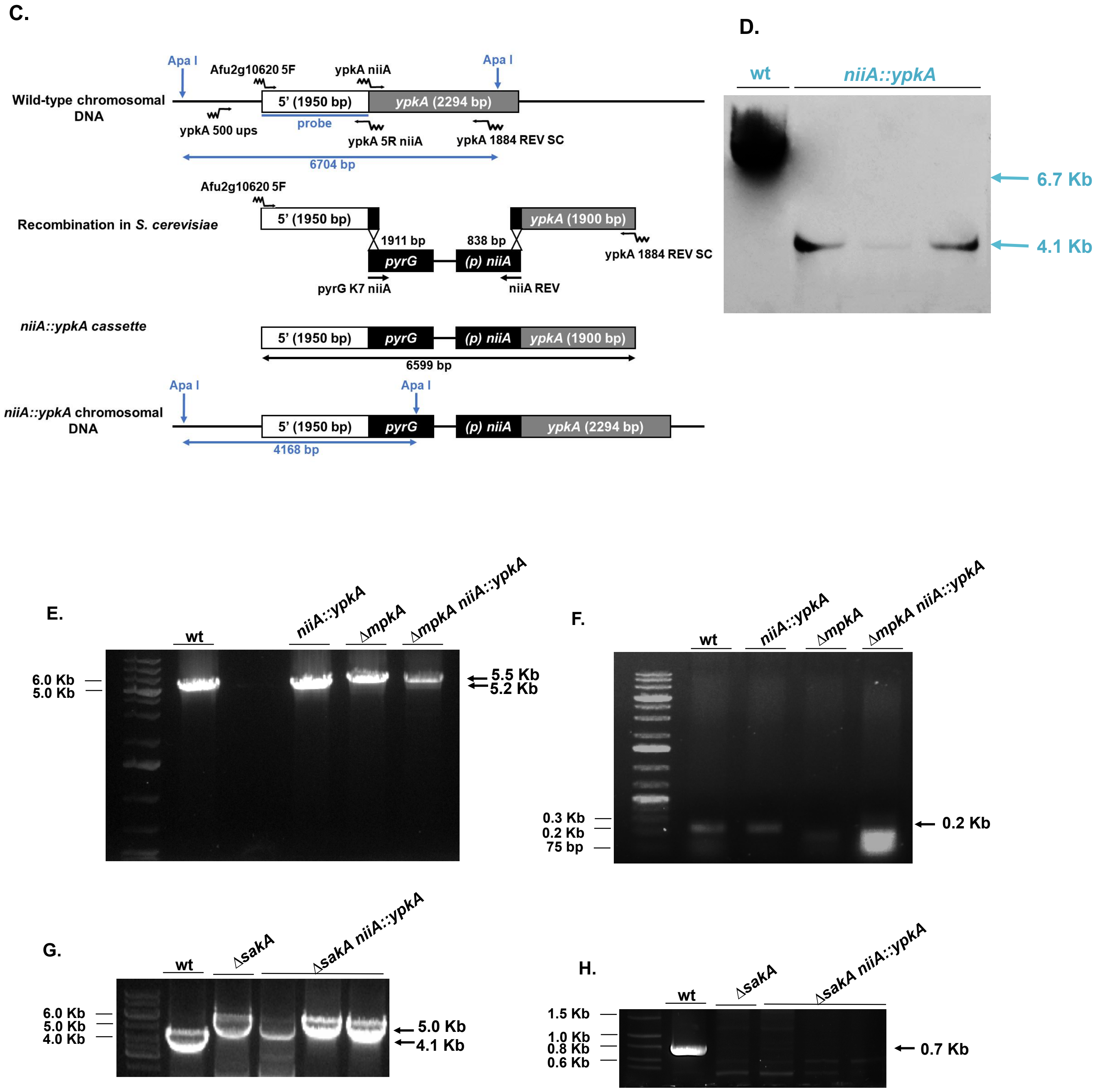

Supplementary Figure 1. Continuation

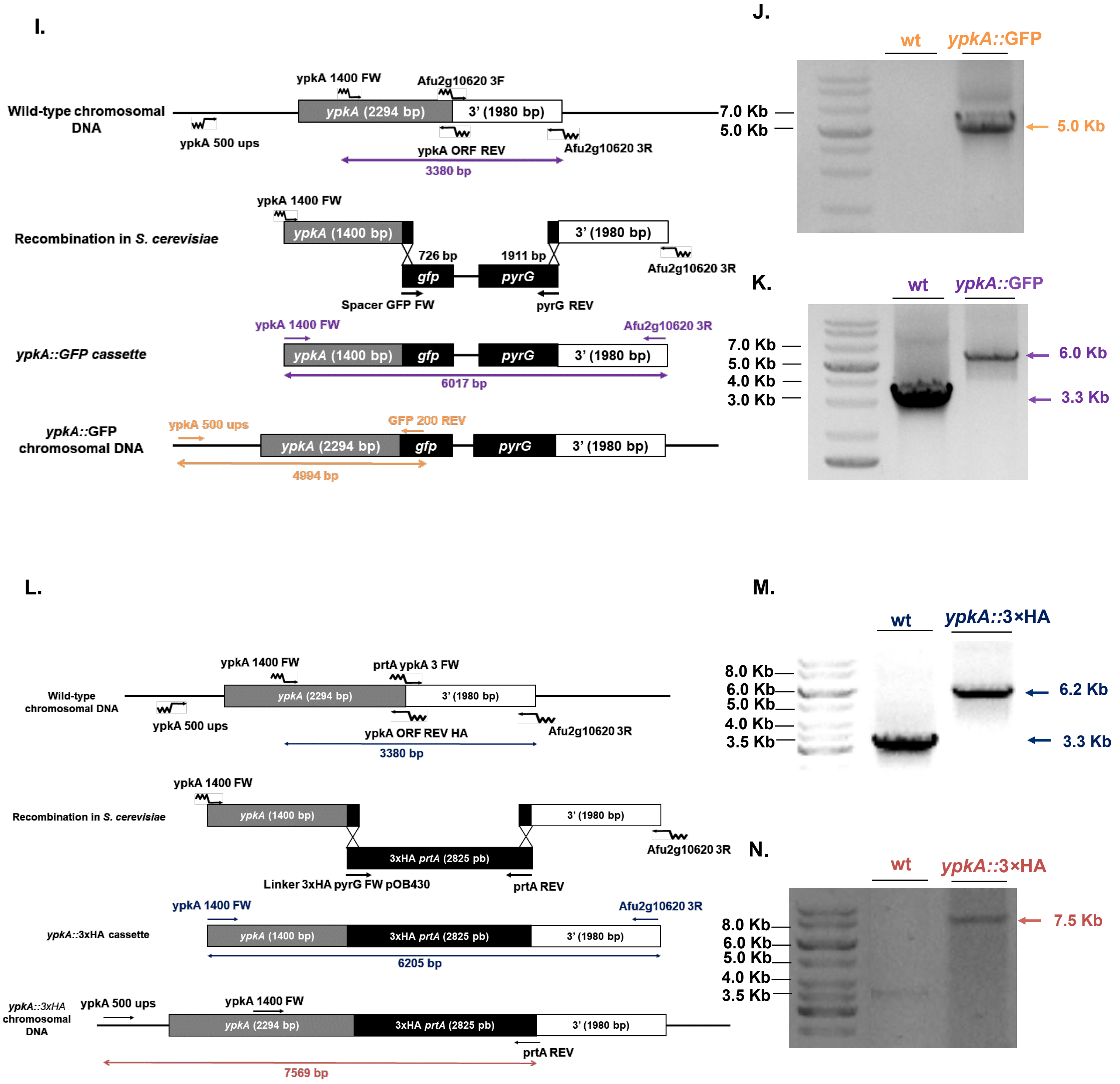

Supplementary Figure 1. Continuation

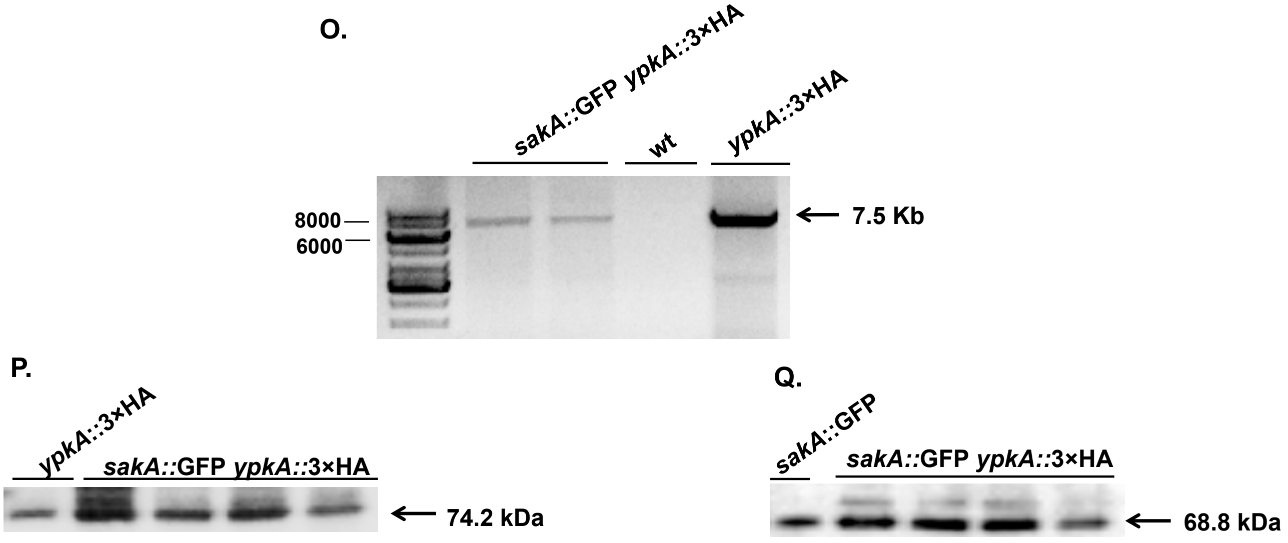

Supplementary Figure 1. Continuation

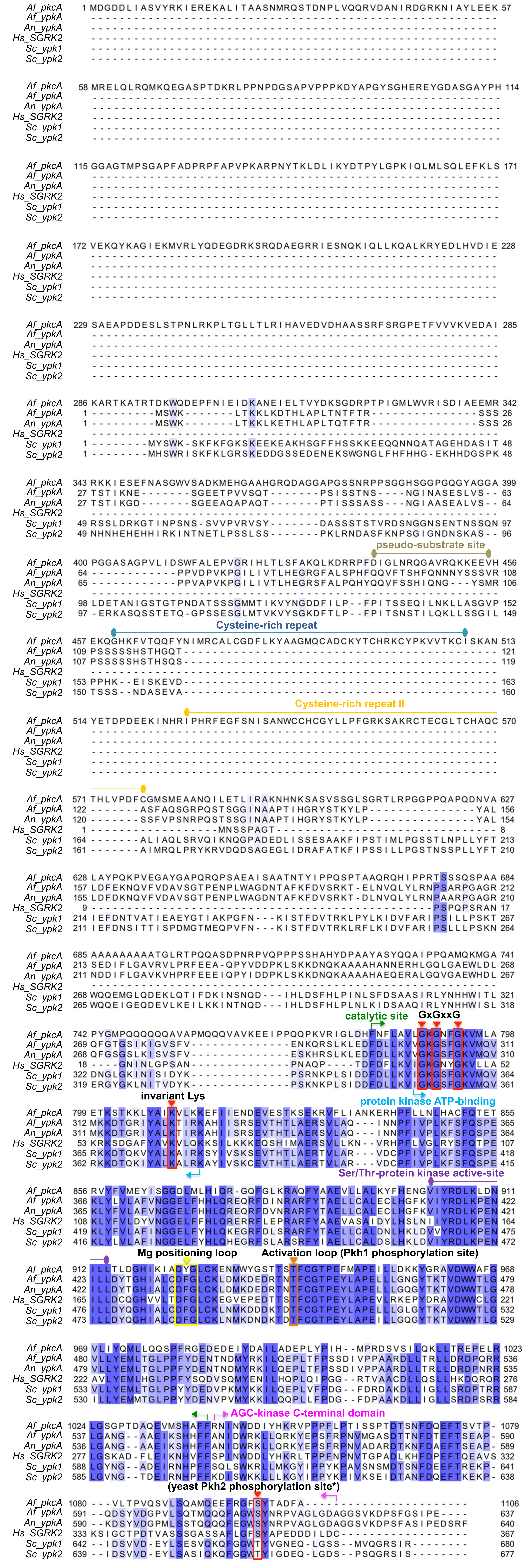

Supplementary Figure S2. Comparison of the deduced amino acid sequences of YpkA in *A. fumigatus* (*Af_ypkA*; Afu2g10620) with the sequences of *S. cerevisiae* Ypk1 (*Sc_ypk1*; YKL126W) and Ypk2 (*Sc_ypk2;* YMR104C), *A. nidulans* YpkA (An_ypkA; AN5973), human Serum Glucocorticoid-Regulated Kinase 2 (*Hs_SGRK2;* NP_001186193.1) and *A. fumigatus* PkcA (*Af_pkcA*; Afu5g11970). Identic residues are depicted in shaded blue boxes. Green, blue and pink arrows showed in opposite orientation indicate the kinase catalytic site, the ATP biding site and the AGC-kinase C-terminal domain, respectively. GxGxxG sequence, which is a structural hallmark of protein kinases and nucleotide binding proteins, and the invariant Lys, which structures the enzyme for phosphoryl-transfer (and is generally mutated to generate kinase-inactive mutants), are shown by the red arrowheads. Domains are based on the overall structure of AGC kinases as given by (Casamayor et al., 1999; Roelants et al., 2004; Herrmann et al., 2006; Steinberg, 2008).

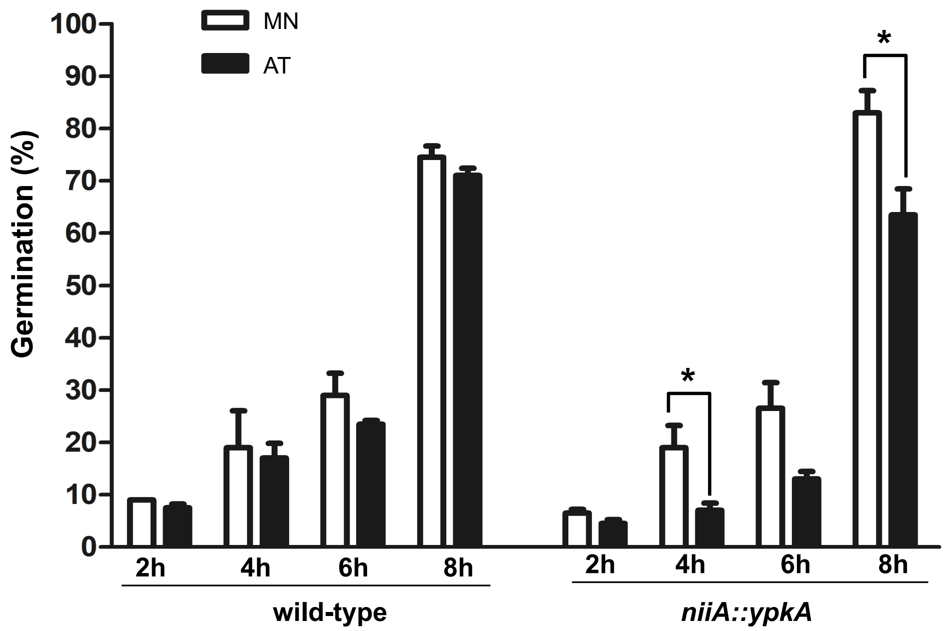

Supplementary Figure 3. Germination of *niiA::ypkA* mutant strain. 1×10^6 conidia of each strain were inoculated in 2 ml of liquid AMM + AT or AMM + MN and incubated at 37°C during 2, 4, 6 and 8 hours before the percentage of germination was evaluated by microscopic inspection. Average ± SD (n =3) are shown (* $p \leq 0.05$, Student's t Test).

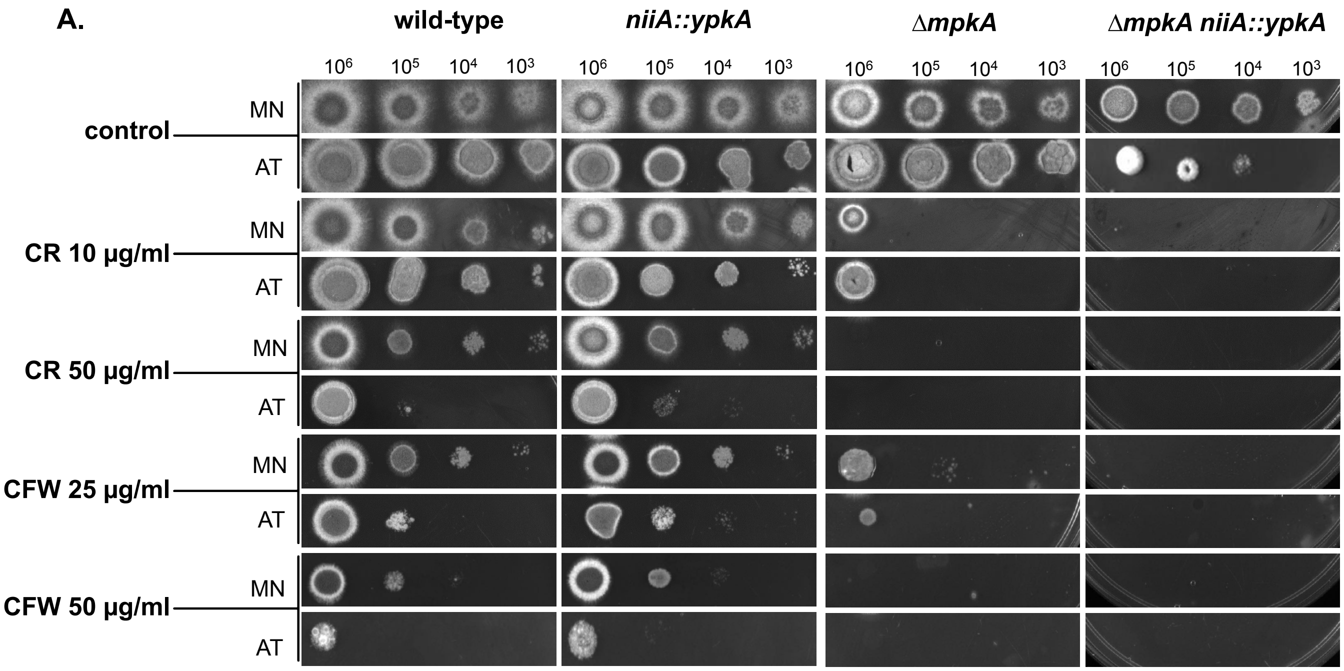

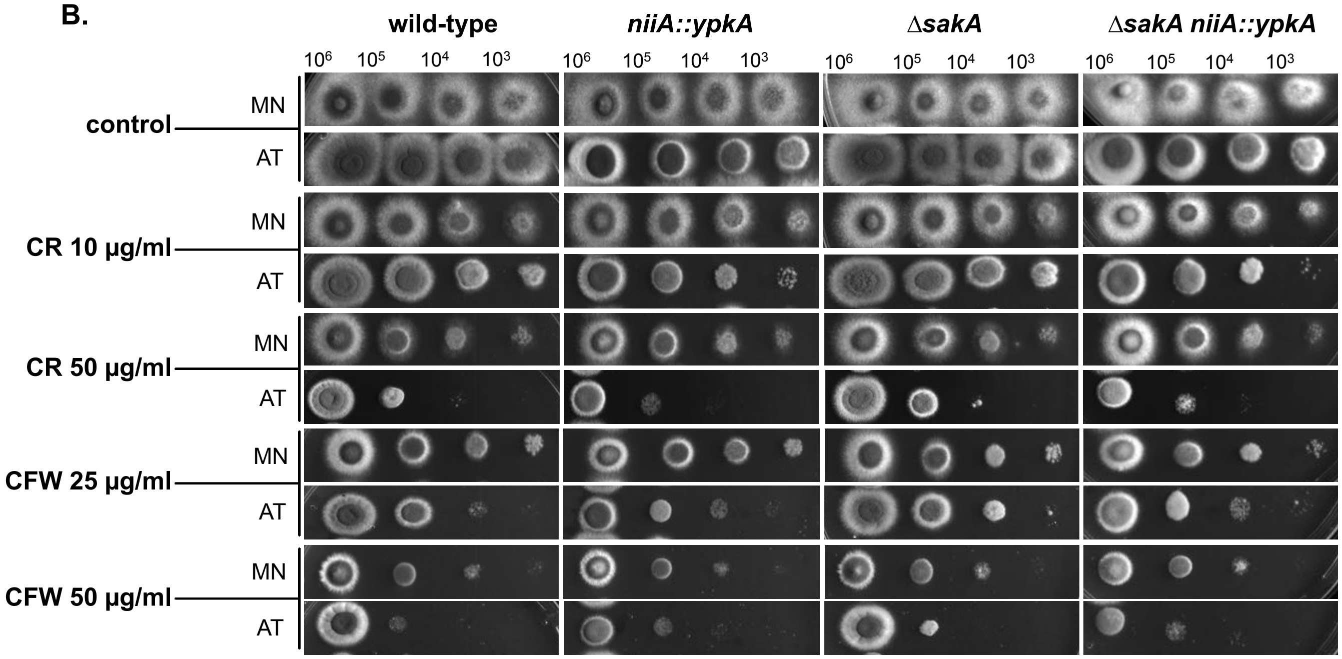

Supplementary Figure 4. Sensitivity of the single and double mutants to cell wall-disturbing compounds. **(A-B)** The indicated number of conidia of each strain were inoculated onto solid AMM plates supplemented with magnesium nitrate (MN) or ammonium tartrate (AT) containing different concentrations of Congo Red (CR) and Calcofluor White (CFW). The plates were incubated for 48 hours at 37ºC.

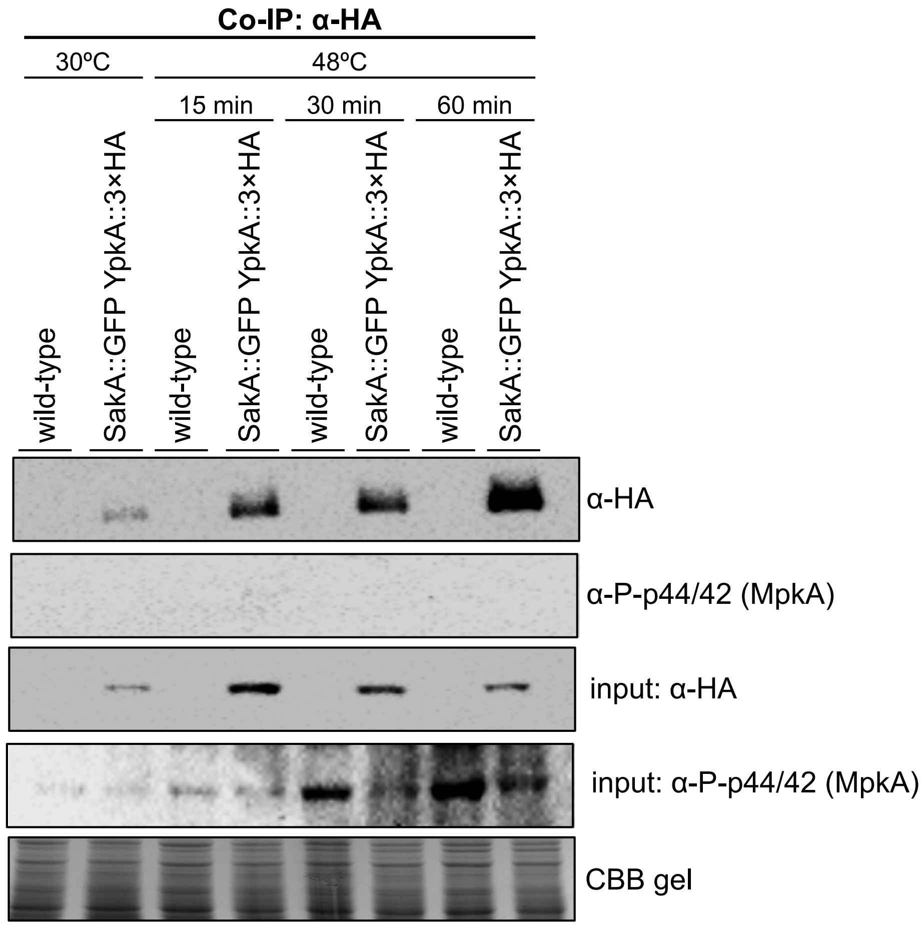

Supplementary Figure 5. YpkA and MpkA do not physically interact *in vivo* during heat shock stress. The wild-type and *sakA*::GFP *ypkA*::3×HA strains were used in the Co-IP assay. Strains were grown at 30°C (24 hours) and subsequently exposed to heat shock at 48°C for the indicated times. Dynabeads Protein A were incubated with monoclonal α-HA antibody and used to immunoprecipitate YpkA::3×HA. Co-immunoprecipitated proteins were investigated by Western blot analysis using α-HA and α-P-p44/42 (MpkA) antibodies. The Coomassie Brilliant Blue (CBB) stained gel was used as an additional input sample control.

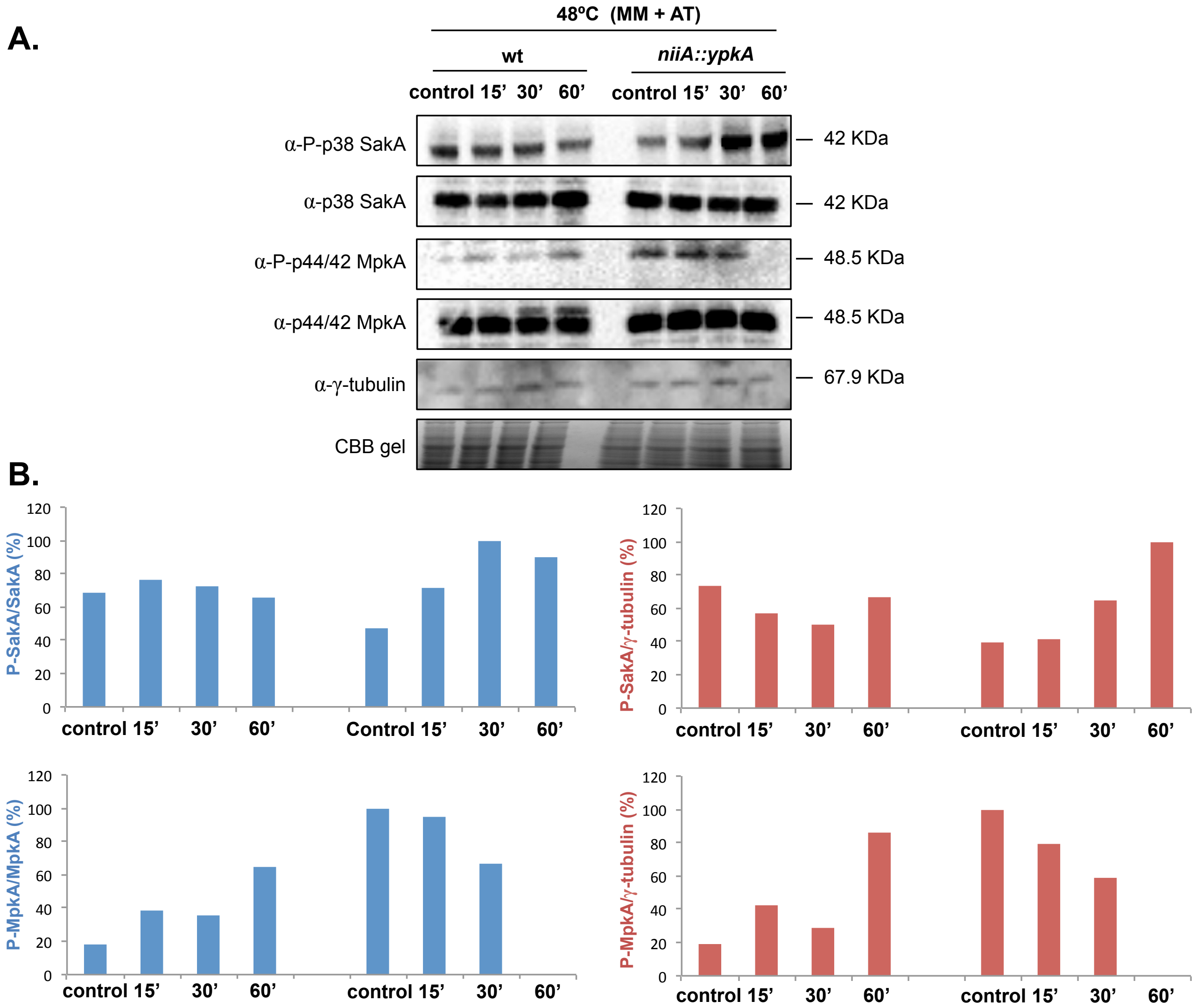

Supplementary Figure 6. Western blotting assay of SakA and MpkA phosphorylation in response to heat shock. **(A)** The wild-type and *niiA::ypkA* strains were grown in AMM + AT and exposed to heat shock at 48ºC for 15, 30 or 60 minutes. The control was left untreated. α-phospho-p38 and α-p38 antibodies were used to detect the phosphorylation of SakA and total SakA, respectively. α-phospho-p44/42 and α-p44/42 antibodies were used to detect the phosphorylation of MpkA and total MpkA, respectively. α-γ-tubulin antibody was used as a loading control. **(B)** Signal intensities were quantified using ImageJ software, and the percentage ratios of p-SakA/SakA, p-SakA/γ-tubulin, p-MpkA/MpkA and p-MpkA/γ-tubulin were calculated in comparison to the untreated control of each strain (blue and red lower graphs). A Coomassie Brilliant Blue (CBB) stained gel of the protein extract served as an additional loading control.

References

Altwasser, R., Baldin, C., Weber, J., Guthke, R., Kniemeyer, O., Brakhage, A.A., et al. (2015). Network Modeling Reveals Cross Talk of MAP Kinases during Adaptation to Caspofungin Stress in Aspergillus fumigatus. *PLoS One* 10(9), e0136932. doi: 10.1371/journal.pone.0136932.

Bruder Nascimento, A.C., Dos Reis, T.F., de Castro, P.A., Hori, J.I., Bom, V.L., de Assis, L.J., et al. (2016). Mitogen activated protein kinases SakA(HOG1) and MpkC collaborate for Aspergillus fumigatus virulence. *Mol Microbiol* 100(5), 841-859. doi: 10.1111/mmi.13354.

Casamayor, A., Torrance, P.D., Kobayashi, T., Thorner, J., and Alessi, D.R. (1999). Functional counterparts of mammalian protein kinases PDK1 and SGK in budding yeast. *Curr Biol* 9(4), 186-197.

Chaveroche, M.K., Ghigo, J.M., and d'Enfert, C. (2000). A rapid method for efficient gene replacement in the filamentous fungus Aspergillus nidulans. *Nucleic Acids Res* 28(22), E97.

da Silva Ferreira, M.E., Kress, M.R., Savoldi, M., Goldman, M.H., Hartl, A., Heinekamp, T., et al. (2006). The akuB(KU80) mutant deficient for nonhomologous end joining is a powerful tool for analyzing pathogenicity in Aspergillus fumigatus. *Eukaryot Cell* 5(1), 207-211.

Fernandez-Abalos, J.M., Fox, H., Pitt, C., Wells, B., and Doonan, J.H. (1998). Plant-adapted green fluorescent protein is a versatile vital reporter for gene expression, protein localization and mitosis in the filamentous fungus, Aspergillus nidulans. *Mol Microbiol* 27(1), 121-130.

Herrmann, M., Sprote, P., and Brakhage, A.A. (2006). Protein kinase C (PkcA) of Aspergillus nidulans is involved in penicillin production. *Appl Environ Microbiol* 72(4), 2957-2970.

Malavazi, I., and Goldman, G.H. (2012). Gene disruption in Aspergillus fumigatus using a PCR-based strategy and in vivo recombination in yeast. *Methods Mol Biol* 845, 99-118. doi: 10.1007/978-1-61779-539-8_7.

Roelants, F.M., Torrance, P.D. and Thorner, J., (2004) Differential roles of PDK1- and PDK2-phosphorylation sites in the yeast AGC kinases Ypk1, Pkc1 and Sch9. *Microbiology* 150, 3289-3304. doi: 10.1099/mic.0.27286-0

Sievers, F., Wilm, A., Dineen, D., Gibson, T.J., Karplus, K., Li, W., et al. (2011). Fast, scalable generation of high-quality protein multiple sequence alignments using Clustal Omega. *Mol Syst Biol* 7, 539. doi: 10.1038/msb.2011.75.

Sobko, A. (2006). Systems biology of AGC kinases in fungi. *Sci STKE* 2006(352), re9. doi: 10.1126/stke.3522006re9.

Steinberg, S.F. (2008). Structural basis of protein kinase C isoform function. *Physiol Rev* 88(4), 1341-1378. doi: 10.1152/physrev.00034.2007.

Teepe, A.G., Loprete, D.M., He, Z., Hoggard, T.A., and Hill, T.W. (2007). The protein kinase C orthologue PkcA plays a role in cell wall integrity and polarized growth in Aspergillus nidulans. *Fungal Genet Biol* 44(6), 554-562. doi: 10.1016/j.fgb.2006.10.001.

Valiante, V., Jain, R., Heinekamp, T., and Brakhage, A.A. (2009). The MpkA MAP kinase module regulates cell wall integrity signaling and pyomelanin formation in Aspergillus fumigatus. *Fungal Genet Biol* 46(12), 909-918. doi: 10.1016/j.fgb.2009.08.005.

Waterhouse, A.M., Procter, J.B., Martin, D.M., Clamp, M., and Barton, G.J. (2009). Jalview Version 2--a multiple sequence alignment editor and analysis workbench. *Bioinformatics* 25(9), 1189-1191. doi: 10.1093/bioinformatics/btp033.
